# Community Mobilization in Mumbai Slums to Improve Perinatal Care and Outcomes: A Cluster Randomized Controlled Trial

**DOI:** 10.1371/journal.pmed.1001257

**Published:** 2012-07-03

**Authors:** Neena Shah More, Ujwala Bapat, Sushmita Das, Glyn Alcock, Sarita Patil, Maya Porel, Leena Vaidya, Armida Fernandez, Wasundhara Joshi, David Osrin

**Affiliations:** 1 Society for Nutrition, Education and Health Action (SNEHA), Urban Health Centre, Chota Sion Hospital, Shahunagar, Dharavi, Mumbai, Maharashtra, India; 2 Centre for International Health and Development, UCL Institute of Child Health, London, United Kingdom; WHO, Switzerland

## Abstract

**Introduction:**

Improving maternal and newborn health in low-income settings requires both health service and community action. Previous community initiatives have been predominantly rural, but India is urbanizing. While working to improve health service quality, we tested an intervention in which urban slum-dweller women's groups worked to improve local perinatal health.

**Methods and Findings:**

A cluster randomized controlled trial in 24 intervention and 24 control settlements covered a population of 283,000. In each intervention cluster, a facilitator supported women's groups through an action learning cycle in which they discussed perinatal experiences, improved their knowledge, and took local action. We monitored births, stillbirths, and neonatal deaths, and interviewed mothers at 6 weeks postpartum. The primary outcomes described perinatal care, maternal morbidity, and extended perinatal mortality. The analysis included 18,197 births over 3 years from 2006 to 2009. We found no differences between trial arms in uptake of antenatal care, reported work, rest, and diet in later pregnancy, institutional delivery, early and exclusive breastfeeding, or care-seeking. The stillbirth rate was non-significantly lower in the intervention arm (odds ratio 0.86, 95% CI 0.60–1.22), and the neonatal mortality rate higher (1.48, 1.06–2.08). The extended perinatal mortality rate did not differ between arms (1.19, 0.90–1.57). We have no evidence that these differences could be explained by the intervention.

**Conclusions:**

Facilitating urban community groups was feasible, and there was evidence of behaviour change, but we did not see population-level effects on health care or mortality. In cities with multiple sources of health care, but inequitable access to services, community mobilization should be integrated with attempts to deliver services for the poorest and most vulnerable, and with initiatives to improve quality of care in both public and private sectors.

**Trial registration:**

Current Controlled Trials ISRCTN96256793

*Please see later in the article for the Editors' Summary*

## Abstract 2

David Osrin and colleagues report findings from a cluster-randomized trial conducted in Mumbai slums; the trial aimed to evaluate whether facilitator-supported women's groups could improve perinatal outcomes.

## Editors' Summary

**Background:**

Substantial progress is being made to reduce global child mortality (deaths of children before the age of 5 years) and maternal mortality (deaths among women because of complications of pregnancy and childbirth)—two of the Millennium Development Goals agreed by world leaders in 2000 to end extreme poverty. Even so, worldwide, in 2010, 7.6 million children died before their fifth birthday and there were nearly 360,000 maternal deaths. Almost all child and maternal deaths occur in developing countries—a fifth of under-five deaths and more than a quarter of neonatal deaths (deaths during the first month of life, which account for two-fifths of all child deaths) occur in India alone. Moreover, most child and maternal deaths are caused by avoidable conditions. Specifically, the major causes of neonatal death—complications of preterm delivery, breathing problems during or after delivery, and infections of the blood (sepsis) and lungs (pneumonia)—and of maternal deaths—hemorrhage (abnormal bleeding), sepsis, unsafe abortion, obstructed labor, and hypertensive diseases of pregnancy—could all be largely prevented by improved access to reproductive health services and skilled health care workers.

**Why Was This Study Done?:**

Experts believe that improvements to maternal and newborn health in low-income settings require both health service strengthening and community action. That is, the demand for better services, driven by improved knowledge about maternal and newborn health (perinatal issues), has to be increased in parallel with the supply of those services. To date, community mobilization around perinatal issues has largely been undertaken in rural settings but populations in developing countries are becoming increasingly urban. In India, for example, 30% of the population now lives in cities. In this cluster randomized controlled trial (a study in which groups of people are randomly assigned to receive alternative interventions and the outcomes in the differently treated “clusters” are compared), City Initiative for Newborn Health (CINH) researchers investigate the effect of an intervention designed to help women's groups in the slums of Mumbai work towards improving local perinatal health. The CINH aims to improve maternal and newborn health in slum communities by improving public health care provision and by working with community members to improve maternal and newborn care practices and care-seeking behaviors.

**What Did the Researchers Do and Find?:**

The researchers enrolled 48 Mumbai slum communities of at least 1,000 households into their trial. In each of the 24 intervention clusters, a facilitator supported local women's groups through a 36-meeting learning cycle during which group members discussed their perinatal experiences, improved their knowledge, and took action. To measure the effect of the intervention, the researchers monitored births, stillbirths, and neonatal deaths in all the clusters and interviewed mothers 6 weeks after delivery. During the 3-year trial, there were 18,197 births in the participating settlements. The women in the intervention clusters were enthusiastic about acquiring new knowledge and made substantial efforts to reach out to other women but were less successful in undertaking collective action such as negotiations with civic authorities for more amenities. There were no differences between the intervention and control communities in the uptake of antenatal care, reported work, rest, and diet in late pregnancy, institutional delivery, or in breast feeding and care-seeking behavior. Finally, the combined rate of stillbirths and neonatal deaths (the extended perinatal mortality rate) was the same in both arms of the trial, as was maternal mortality.

**What Do These Findings Mean?:**

These findings indicate that it is possible to facilitate the discussion of perinatal health care by urban women's groups in the challenging conditions that exist in the slums of Mumbai. However, they fail to show any measureable effect of community mobilization through the facilitation of women's groups on perinatal health at the population level. The researchers acknowledge that more intensive community activities that target the poorest, most vulnerable slum dwellers might produce measurable effects on perinatal mortality, and they conclude that, in cities with multiple sources of health care and inequitable access to services, it remains important to integrate community mobilization with attempts to deliver services to the poorest and most vulnerable, and with initiatives to improve the quality of health care in both the public and private sector.

**Additional Information:**

Please access these Web sites via the online version of this summary at http://dx.doi.org/10.1371/journal.pmed.1001257.

- The United Nations Childrens Fund (UNICEF) works for children's rights, survival, development, and protection around the world; it provides information on the reduction of child mortality (Millennium Development Goal 4); its Childinfo website provides information about all the Millennium Development Goals and detailed statistics about on child survival and health, newborn care, and maternal health (some information in several languages)
- The World Health Organization also has information about Millennium Development Goal 4 and Millennium Development Goal 5, the reduction of maternal mortality, provides information on newborn infants, and provides estimates of child mortality rates (some information in several languages)
- Further information about the Millennium Development Goals is available
- Information on the City Initiative for Newborn Health and its partners and a detailed description of its trial of community mobilization in Mumbai slums to improve care during pregnancy, delivery, postnatally and for the newborn are available
- Further information about the Society for Nutrition, Education and Health Action (SNEHA) is available

## Introduction

The current consensus on improving maternal and newborn survival [1]–[3], particularly in low-income countries, is that perinatal care needs to involve both health service strengthening and community activities [4]–[6]. Recent advances include India's Janani Suraksha Yojana cash incentive for professional obstetric care [7], the development of home-based newborn care as a result of work in rural India [8]–[11], and the success of community mobilization around perinatal issues through women's groups [12],[13]. The balance of supply- and demand-side interventions remains uncertain: community mobilization seems likely to yield greater benefits when mortality rates are high, and a focus on health service quality when they are lower.

Community mobilization in this context has been almost exclusively rural, but urban health is a developing concern [14]. Currently, 30% of India's population is urban and 50 cities have populations of over 1 million [15]. Rural health inequalities are replicated in urban settings [16]–[19], with some important differences. Cities offer many potential sources of health care at a range of prices, and urban health care in India is provided by a burgeoning, largely unregulated private sector and a beleaguered public sector that caters mainly to poorer groups. In this context, both quality of care and choice of an appropriate provider are problematic [20],[21]. Access tends not to be limited by distance and scarcity, but by direct and indirect costs: urban life and available time are intensely monetized and 80% of health care expenditure is out of pocket [22]. Environmental determinants of health such as housing fabric, water, sanitation, and sewage dominate community perceptions of health needs in vulnerable urban communities. Socio-cultural heterogeneity hinders collective action, while an overburdened urban infrastructure struggles to meet growing demand.

Mumbai's City Initiative for Newborn Health began in 2004. A partnership between the Society for Nutrition, Education and Health Action (SNEHA), a non-government organisation (NGO) committed to improving the health of women and children in Mumbai's slums, the Municipal Corporation of Greater Mumbai (MCGM), and University College London aimed to improve maternal and newborn health in slum communities. Planning of India's National Urban Health Mission, analogue of the developing National Rural Health Mission, was underway. An Urban Social Health Activist (USHA) would work in vulnerable communities, but what she would do was undecided. The City Initiative was conceived as operationalizing a combination of supply- and demand-side interventions in the urban context. It combined improvement in municipal health care provision—reinstitution of antenatal clinics at health posts catering to the urban poor, clinical protocols for maternal and newborn care, improved communication between institutions, and consolidation of referral linkages—with community mobilization activities [23].

The community activities took as their model south Asian projects that have worked successfully with groups of rural women to improve perinatal health in their own communities, in a test of their transferability to urban slum populations [12],[13],[24]. The trial was a piece of operational research within the City Initiative. Our objective was to test an intervention in which slum-dweller women's groups discussed perinatal health, improved their knowledge through peer-learning, and developed and implemented local strategies. We examined a range of outcomes, including stillbirth and neonatal mortality rates and antenatal, intrapartum, and postpartum care (Text S1).

## Methods

### Ethics Statement

The activities of the City Initiative for Newborn Health were approved by MCGM Joint Municipal Commissioners in 2004. Given our interest in operational research at the interface between NGO activities and public health evaluation, the idea of a trial was in our minds. However, we were aware of no previous cluster randomised controlled trial (cRCT) involving urban slums, and there were serious doubts about whether it would be possible. Additionally, little was known about maternal and child health in Mumbai slums. For these reasons, in 2005, we set up a perinatal vital registration system in 48 slum communities as part of the City Initiative. The number was chosen because it was large enough to model scalability and would be sufficient if we proceeded to a trial. The surveillance was successful and it became clear that there was scope for achieving a cRCT design. Funding for a trial was received in 2007. Ethical approval was granted in the same year by the Independent Ethics Committee for Research on Human Subjects (Mumbai, reference IEC/06/31), the trial was registered (ISRCTN96256793), and its protocol was published in 2008 (Text S2) [25]. Cluster-level verbal consent for the study was given after community meetings with general practitioners, community-based organizations, NGOs, municipal representatives, political officers of major parties, and social workers.

### Setting and Design

The capital of Maharashtra state, Mumbai has a provisional 2011 census population of 12.4 million, more than half of whom live in slums. About one-fifth of slum homes have a private toilet, 31% of residents have completed 10 y of education, and the total fertility rate is below the replacement threshold at 1.9 [26]. Public sector care is provided by the Municipal Corporation. Private health care is widely available and ranges from specialty hospitals to informal practitioners. A cRCT design was chosen because a group intervention was delivered in communities. Key participants were women who joined groups in 24 intervention clusters, to be compared with 24 control clusters. We implemented the trial in six municipal wards. Clusters included had at least 1,000 households, residents were aware of no plans for resettlement, and cluster separation was wide enough to minimise contamination. We excluded areas with transient communities—large construction gangs, pavement dwellings—and areas for which resettlement was being negotiated.

### Intervention

**Figure 1:**
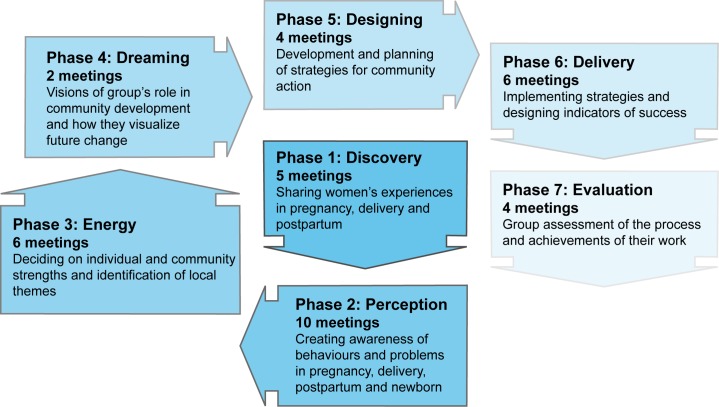
The intervention cycle.

All the human resources involved in community activities were employed by SNEHA. We recruited one full-time facilitator in each intervention cluster of about 1,000 households. This *sakhi* (friend) was a local woman with secondary education and leadership skills, preferably married with children. Her role was to conduct meetings with women, attend planning and supervision meetings, and support group action. After training, she began by profiling her settlement and building rapport with local stakeholders. She also attended regular training on a range of health care topics. Over about 6 mo, she set up ten women's groups, formative work having shown that women's mobility tended to be confined to their own alley. The groups met fortnightly and she met weekly with other *sakhis* and her supervisor. The intervention followed a 36-meeting cycle that was predetermined in general but developed iteratively in detail (Figure 1). There was no set point at which women had to join a group, and women of all ages, pregnant and non-pregnant, were welcome to participate. We took a participatory approach with an emphasis on sharing and peer-learning, rather than on the *sakhi* as an expert resource, and used the change methodology of Appreciative Inquiry to focus on the positive and to build energy for action through identification of the strengths of participants, their families, and neighbourhoods [27]. Appreciative approaches have gained traction as an alternative to problem-focused approaches [28],[29], although empirical evaluation in terms of health outcomes has been limited. Each step was simulated in *sakhis'* weekly meetings and supported by supervisors. The emphasis was on knowing what services were available, choosing appropriate perinatal health care, understanding best practice, and negotiating optimal care with family and providers.

### Outcomes

Routine and emergency antenatal, intrapartum, and postnatal care was documented, as well as illness in mother or baby. Previous trials in rural settings had shown no effects on stillbirth rates. In an urban setting with high levels of institutional delivery, we decided to present both stillbirth (death of an infant before or during delivery, per thousand births) and neonatal mortality (death of a live-born infant within the first 28 d of life, per thousand live births) rates.

### Sample Size and Allocation

Using data collected in the pre-trial year, with a two-tailed 5% significance level, a conservative coefficient of variation between clusters (*k*) value of 0.3, and the recruitment numbers achieved, the trial had 80% power to detect a reduction in stillbirths from 12 to 7 per thousand, in neonatal deaths from 20 to 12 per thousand, and in extended perinatal mortality from 29 to 20 per thousand [30]. Through municipal documents, surveys, discussions with key informants, and site visits, we identified 92 slum clusters. In a transparent process, social workers external to the trial drew lots to select 48 in blocks of eight per ward, and then to allocate four clusters per block to the intervention. We chose this method because of our emphasis on participation and demystification of research. The nature of the intervention precluded allocation concealment.

### Data Collection and Management

*Sakhis* recorded details of each meeting they facilitated, quantitative elements of which were entered into an electronic database. Supervisors recorded their observations of meetings and training and feedback sessions. A process evaluation officer attended and documented weekly meetings and interviewed *sakhis*, group members and non-members, and local stakeholders. We were able to draw on attendance and content data for 5,253 women's group meetings, 120 meetings observed by supervisors, summaries of 150 valuation meetings, ten focus group discussions, seven sets of questionnaires, seven role-play exercises, and interviews with 39 local stakeholders. Group attendance was documented by *sakhis* and supplemented with interview data collected by the process evaluation officer. We used the attendance data to identify group members in all 24 intervention clusters who had attended at least 15 meetings, and whose groups had reached the latter stages of the meeting cycle. We asked these women what they had done in terms of outreach, and subsequently categorized their responses.

The trial covered an estimated population of 283,000. The community-based vital surveillance system across the 48 clusters was based on previous models [12],[31]. Live births, stillbirths, neonatal deaths, and maternal deaths were identified by 99 locally resident women, each covering an average 600 households and receiving INR 50 (US$1.11) per identification. Every event was confirmed by one of 12 interviewers, who took verbal consent for a comprehensive interview at around 6 wk after delivery (Text S3). In the event of a death, one of six supervisors conducted verbal autopsy. Because of a fall in documented births later in the trial, we did a retrospective census of births and deaths in all 48 clusters. After the trial finished, we gave lists of all documented births and deaths in the preceding year to the field investigators, and to fieldworkers from other SNEHA projects. These agents checked house to house for any births or deaths that might not have been identified. The process identified 41 previously undocumented births, but no stillbirths or neonatal deaths.

We had an ethical responsibility to recommend that unwell mothers or infants visit a health facility, and to expedite care in emergencies. Records of events and completed questionnaires were subject to systematic and random checks for accuracy and completeness, both in the field and during entry into an electronic database (Access; Microsoft Corporation). Information provided by participants remained confidential and outputs did not include their names.

A steering group of external experts met in the first and second years and reviewed the design, intervention and recruitment. A Data Monitoring Committee met in April 2009, looked at data from the first 2 y, and recommended stopping the intervention after its cycles were complete and finishing the data collection as planned. Data collection continued until mid-2010 in order to pick up outcomes from all births to end-September 2009. It included a retrospective census to ensure data completeness. A further meeting in March 2011 considered the analyses and discussed presentation and publication.

### Statistical Methods

The analysis used records of individual births and was by intention to treat at cluster level. Analysts were blind to allocation. For potential effects on health care, morbidity, and mortality, we did multivariable logistic regression with random effects grouped on cluster, comparing outcomes in intervention and control arms and adjusting for covariates by including them in the models. Quadrature checks confirmed the applicability of this approach. The Data Monitoring Committee recommended adjustment for socioeconomic status and for Muslim faith. Socioeconomic status was described by a household asset score based on standardised weights for the first component of a principal components analysis [32],[33]. This adjustment made no appreciable difference to the findings, and we present unadjusted odds ratios and 95% confidence intervals. There were differences in the mortality findings after adjustment, and we present unadjusted models, along with models adjusted for cluster mean household asset score at baseline, cluster proportion of Muslim respondents at baseline, and baseline cluster mortality rate [34].

The primary analysis compared records from intervention and control arms collected over 3 y. We did four pre-specified ancillary analyses using identical methods on successively filtered datasets: a comparison of outcomes in the latter 2 y of the trial, a comparison that included only women who had lived in a cluster for at least a year, a comparison of outcomes stratified by socioeconomic status, and a comparison of women who had been group members with women in intervention clusters who had not been members. Analyses were done in Stata 11. Causes of perinatal death based on verbal autopsies were assigned independently by two physicians, using an international classification [35]. Discordance was resolved by a pediatrician (DO).

## Results

### Process

The conceptual premise of the model was that facilitation of women's groups would lead to changes in members' knowledge and behaviour, with diffusion of influence to other local women. When the project began, women expressed a need for information on health and health care. Although slum women's groups (*Mahila mandals*) and action groups existed in some clusters, they tended to focus on single issues on an ad hoc basis. *Sakhis* formed 244 groups, a median ten per cluster. Groups were based in individual alleys because members were often reluctant to circulate, and the small size of homes restricted attendance. Other considerations included women's need to do piecework and to care for their families. A mean five women attended each meeting (range 2–20) and individual women attended 15 meetings each (range 1–50).

Group members were enthusiastic about acquiring new knowledge. They made substantial efforts to reach out to other local women: a sample of 235 members helped 1,372 other distinct women so that, on average, one woman reached out to six others, particularly by providing them with information on antenatal and newborn care, suggesting that they visit health providers, and occasionally accompanying them. Achieving collective action was, however, more challenging. Key activities undertaken by groups were attempts to create local awareness, extending support to other women, negotiating with civic authorities for amenities, and mobilizing and sharing resources. One group succeeded in getting the municipal corporation to cover local sewage channels, but efforts were generally more individual than collective and involved women's own families and neighbourhoods. Their desire for knowledge was tempered by time pressure and immediate concerns such as insecurity of tenure, which limited their inclination to get involved in wider action. There was attrition in the numbers of groups and membership in the later phases that attempted to broker collective strategizing. Membership was informal and withdrawal easy when participants felt that the commitment required would be onerous. 150 groups were sustained to the end of the cycle and member numbers dropped from an initial 2,948 to 656.

### Recruitment

**Figure 2:**
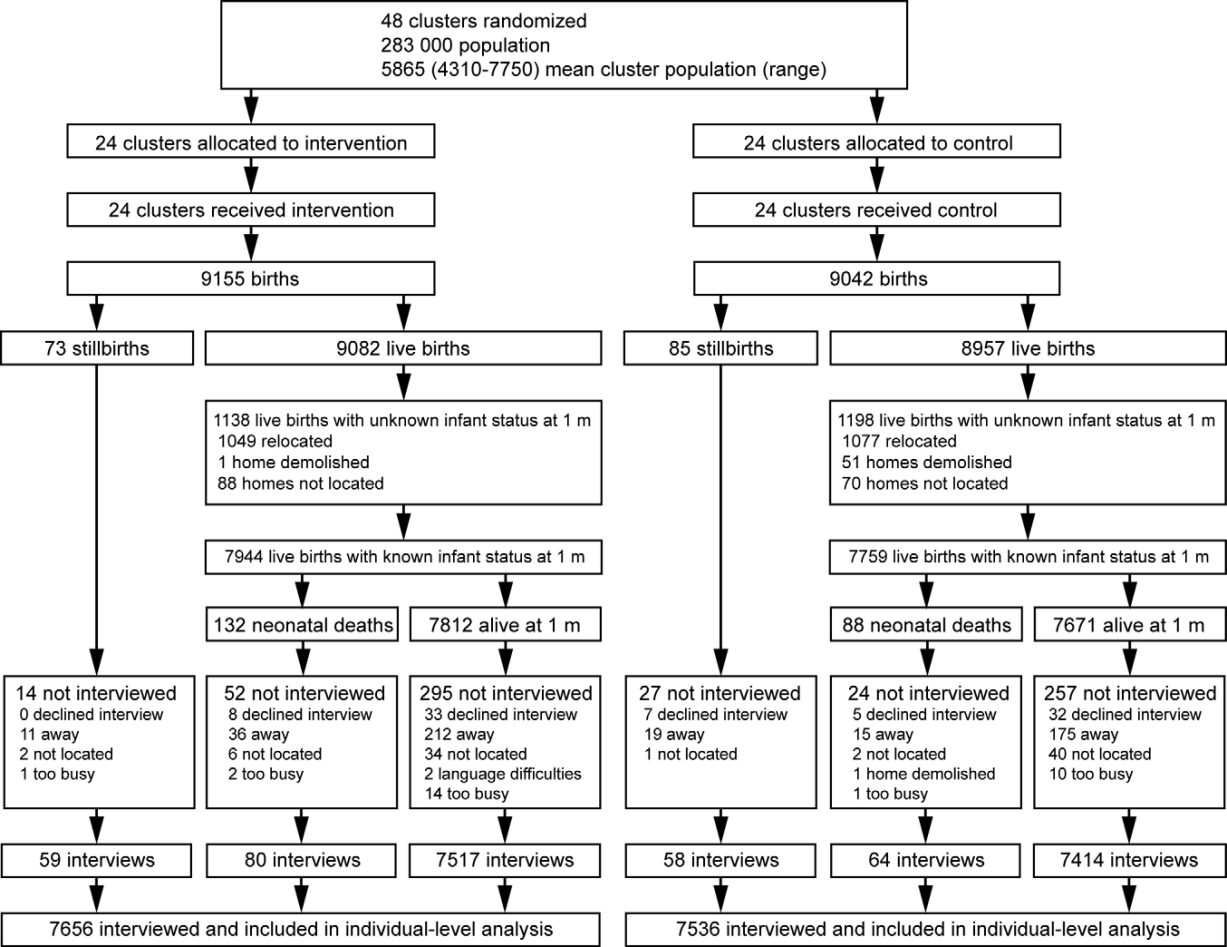
Trial profile.

The trial ran from 1st October 2006 to 30th September 2009. We designated the pre-trial year of data collection a baseline period, leaving 3 complete years for the evaluation of effect. Figure 2 shows the trial profile.

### Baseline Comparison

Table 1 summarizes cluster size and participant characteristics. There were insufficient births in nine clusters and we expanded their perimeters for subsequent years. Two clusters were reduced because of excess births. Numbers of households, population, and births were similar in intervention and control arms. The crude birth rate was 23 per 1,000. Mean maternal age was 24.2 y (standard deviation [SD] 4.05) in the intervention arm and 24.6 y (SD 4.33) in the control arm. A higher proportion of households in the control arm followed Islam (58%, compared with 33%). Control clusters had slightly more of the poorest quintile of women than intervention clusters (21% compared with 20%), but also of the least poor (23% compared with 17%). Health care practices were similar in intervention and control arms (unpublished data). Baseline stillbirth rates were 12.5 per thousand live births in both arms (42/3,347 in intervention and 42/3,338 in control). Neonatal mortality rates were 22.3 and 18.6 (63/2,845 in intervention and 50/2,712 in control).

**Table 1:** Cluster size and characteristics of women interviewed at 6 wk postpartum, comparing allocation groups in the 3 trial years.

Cluster Size and Characteristics	Intervention	Percent	Control	Percent
**Cluster size**				
Households: median (range)	1,191 (800–1,793)	**—**	1,173 (862–1,550)	**—**
Population: median (range)	5,917 (4,000–8,965)	**—**	5,863 (4,310–7,750)	**—**
**Characteristics of women**	7,656	(100.0)	7,536	(100.0)
Age				
<20 y	684	(8.93)	642	(8.52)
20–29 y	6,069	(79.27)	5,816	(77.18)
30 y and over	896	(11.70)	1,065	(14.13)
Unknown	7	(0.09)	13	(0.17)
Education				
No schooling	1,817	(23.73)	2,117	(28.09)
Primary	395	(5.16)	402	(5.33)
Secondary	4,652	(60.76)	4,307	(57.15)
Higher	792	(10.34)	710	(9.42)
Religion				
Hindu	4,423	(57.77)	2,925	(38.81)
Muslim	2,492	(32.55)	4,399	(58.37)
Buddhist	550	(7.18)	142	(1.88)
Other	191	(2.49)	70	(0.93)
Duration of residence				
<1 y	1,618	(21.13)	1,515	(20.10)
1–5 y	4,036	(52.72)	3,778	(50.13)
>5 y	2,000	(26.12)	2,241	(29.74)
Missing data	2	(0.03)	2	(0.03)
Asset score quintile				
1 (poorest)	1,506	(19.67)	1,514	(21.34)
2	1,505	(19.66)	1,487	(19.36)
3	1,645	(21.49)	1,426	(18.48)
4	1,698	(22.18)	1,552	(17.81)
5 (least poor)	1,302	(17.01)	1,557	(23.01)
Parity				
One	2,691	(35.15)	2,380	(31.58)
Two	2,255	(29.45)	2,035	(27.00)
Three	1,457	(19.03)	1,433	(19.02)
Four	721	(9.42)	817	(10.84)
Five or more	532	(6.95)	871	(11.56)

### Impact Evaluation

**Figure 3:**
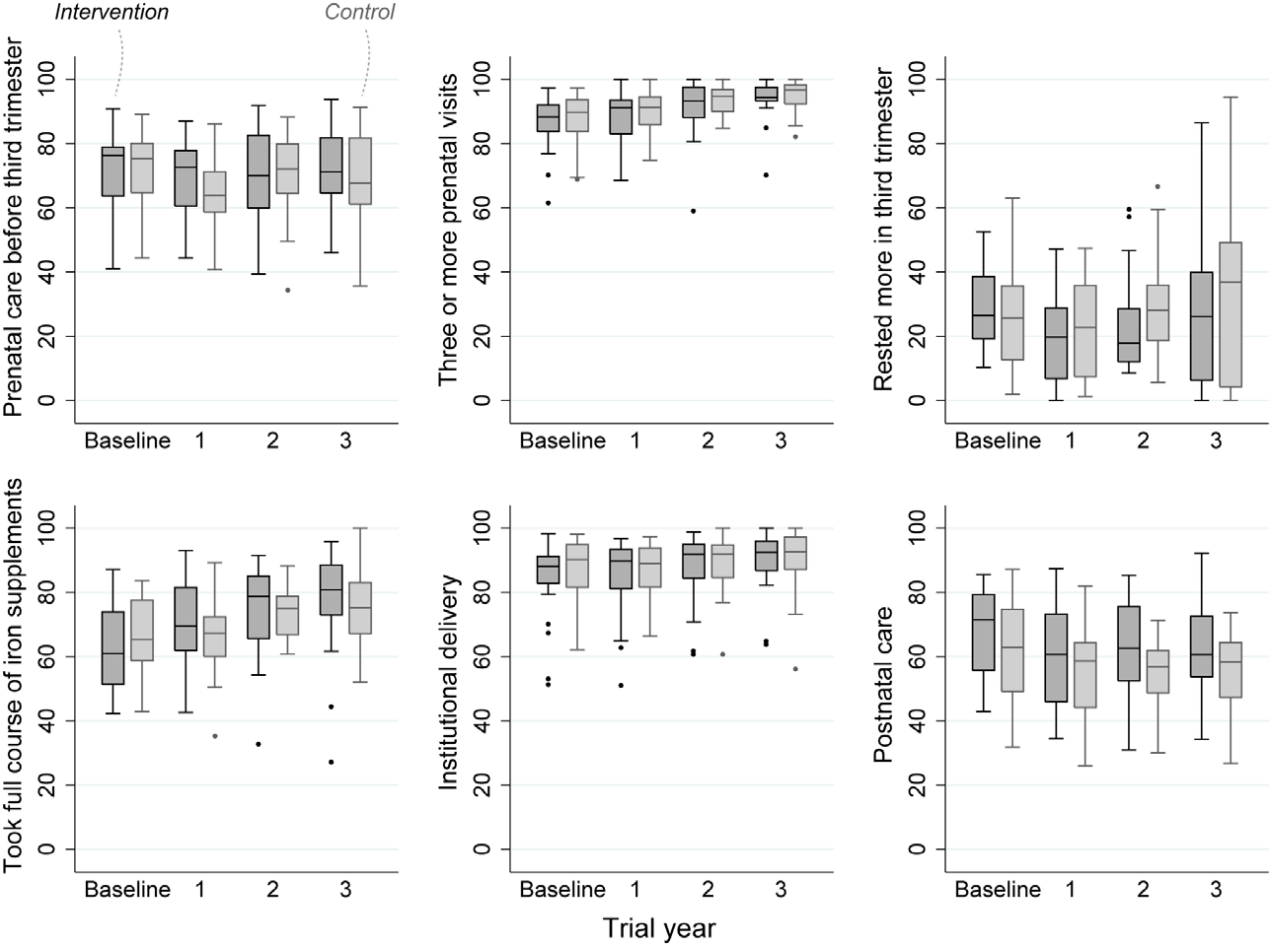
Perinatal care practices, percentage in each allocation group, by trial year.

**Figure 4:**
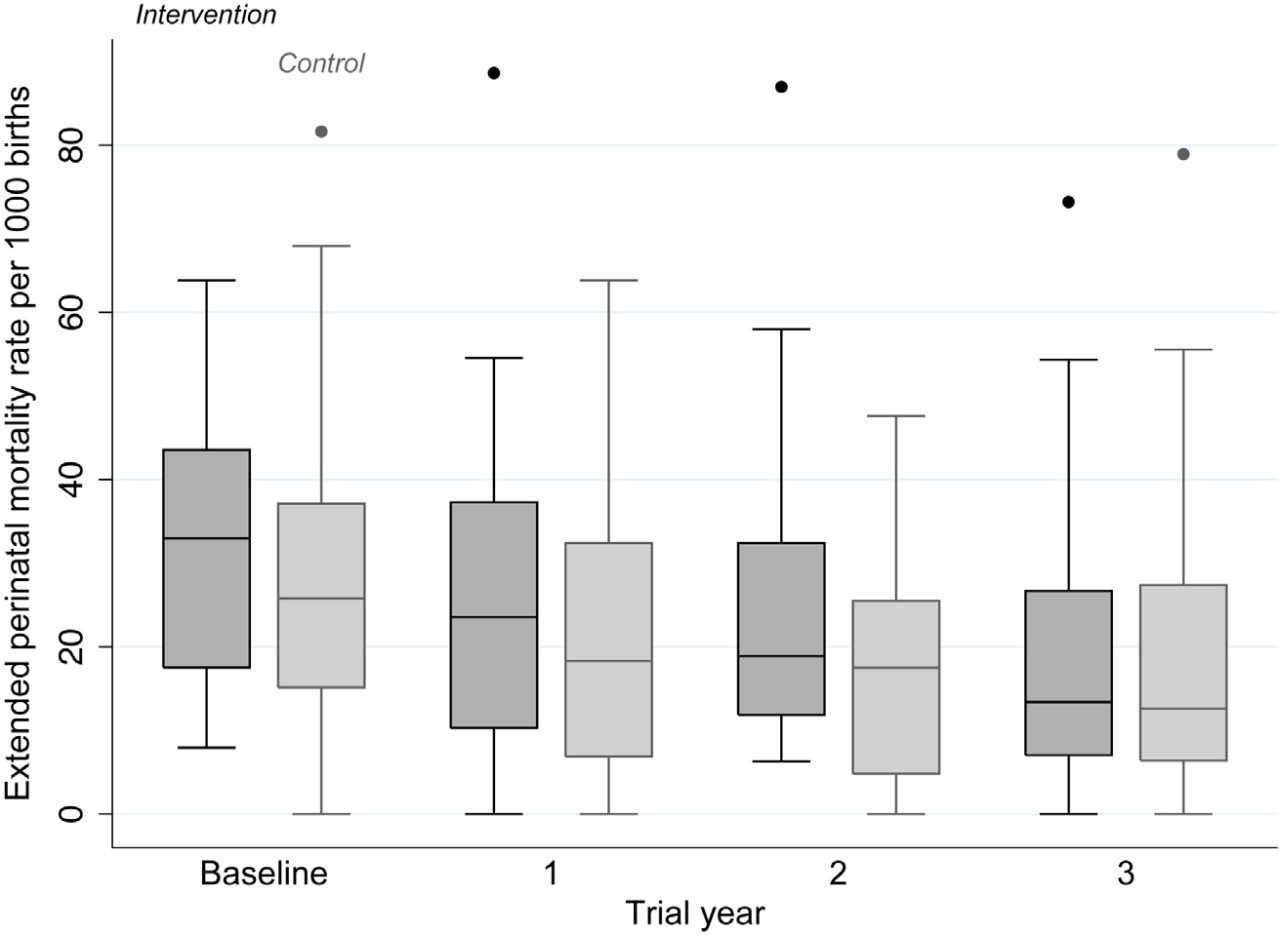
Extended perinatal mortality rate in each allocation group, by trial year.

The intention-to-treat analysis included 18,197 births over 3 y, a median 379 births per cluster (interquartile range [IQR] 263–480). For analysis of neonatal mortality rates, the vital status at 1 mo of 15,703 live-born infants was known. Analysis of other outcomes was based on detailed interviews with 15,192 women. We achieved interviews after 84% of births in the intervention and 83% in the control arm. Table 2 presents the findings for behavioural and morbidity outcomes, and Figure 3 time-series examples. We found no differences between intervention and control arms in uptake of antenatal care, reported work, rest and diet in later pregnancy, institutional delivery, early and exclusive breastfeeding, or care-seeking for maternal or neonatal problems. The occurrence of serious antenatal symptoms (premature rupture of membranes, antepartum hemorrhage, cessation of fetal movements, or maternal seizures) was less common in the intervention arm. Table 3 presents the mortality findings. The stillbirth rate was lower in the intervention arm, although this finding was only significant after adjustment for religion, socioeconomic status, and baseline cluster stillbirth rate (adjusted odds ratio 0.66, 95% CI 0.46–0.93). The neonatal mortality rate was higher in the intervention arm, but non-significantly after similar adjustment (1.42, 0.99–2.04). A combination of both these outcomes, the extended perinatal mortality rate, did not differ between arms (1.01, 0.78–1.31); Figure 4 shows an annual boxplot. There were 20 maternal deaths in the intervention and 24 in the control group, a combined maternal mortality ratio of 244 per 100,000 live births. We are aware of no harms associated with the intervention.

**Table 2:** Primary analysis of health care and morbidity outcomes over 3 y, comparing intervention and control arms.

Outcomes	Intervention	Percent	Control	Percent	OR	(95% CI)
Births	7,656	(100.00)	7,536	(100.00)		
First antenatal visit before 3rd trimester	5,306	(69.31)	4,949	(65.67)	1.13	(0.84–1.51)
Antenatal care in public sector	3,229	(42.18)	3,308	(43.90)	1.03	(0.75–1.41)
Three or more antenatal care visits	6,950	(90.78)	6,932	(91.99)	0.85	(0.57–1.27)
Three packets of iron supplements	5,639	(73.65)	5,372	(71.30)	1.18	(0.87–1.60)
Rested more in 3rd trimester	1,839	(24.05)	2,089	(27.77)	0.74	(0.47–1.19)
Worked less in 3rd trimester	2,303	(30.13)	2,261	(30.06)	0.80	(0.48–1.34)
Ate more in 2nd and 3rd trimesters	1,122	(14.66)	884	(11.73)	1.20	(0.60–2.39)
Sentinel antepartum symptom (leaking of waters, vaginal bleeding, baby not moving, convulsion or loss of consciousness)	408	(5.33)	732	(9.71)	0.60	(0.38–0.94)
Sought clinical care for trigger symptom within 24 h	83	(20.34)	84	(11.48)	1.60	(0.84–3.03)
Institutional delivery	6,602	(86.23)	6,573	(87.22)	0.92	(0.58–1.47)
At public maternity home	831	(10.85)	721	(9.57)	1.20	(0.47–3.08)
At public tertiary hospital	1,157	(15.11)	967	(12.83)	0.97	(0.43–2.15)
At private hospital	2,113	(27.60)	2,369	(31.44)	0.77	(0.55–1.09)
Postnatal check	4,616	(60.29)	4,046	(53.69)	1.35	(1.00–1.81)
Infant sex female	3,514	(46.75)	3,513	(47.38)	0.97	(0.91–1.04)
Breastfed within 24 h	6,198	(82.75)	6,077	(82.40)	1.10	(0.89–1.36)
Exclusively breastfed for at least 28 d	5,297	(70.47)	4,943	(66.67)	1.21	(0.95–1.54)
Infant BCG	6,932	(92.22)	6,803	(91.76)	1.14	(0.72–1.79)
Any newborn problem	2,590	(33.83)	2,566	(34.05)	1.00	(0.83–1.22)
Sought clinical care for specified newborn illness within 24 h	456	(17.61)	468	(18.24)	0.92	(0.73–1.17)

Logistic regression with random effect for cluster. Data collected by questionnaire at about 6 wk postpartum.

BCG, Bacille Calmette-Guerin; OR, odds ratio.

**Table 3:** Primary analysis of mortality outcomes over 3 y, comparing intervention and control arms.

Mortality Outcomes	Intervention	Control	Unadjusted OR (95% CI)	Adjusted for Baseline Mortality Rate OR (95% CI)	Adjusted for Baseline Mortality Rate, Muslim Faith, and Asset Score OR (95% CI)
Stillbirths	73/9,155	85/9,042	—	—	—
Rate per 1,000	7.97	9.40	0.86 (0.60–1.22)	0.86 (0.60–1.21)	0.66 (0.46–0.93)
Neonatal deaths	132/7,944	88/7,759	—	—	—
Rate per 1,000	16.62	11.34	1.48 (1.06–2.08)	1.44 (1.03–2.01)	1.42 (0.99–2.04)
Extended perinatal deaths	205/9,155	173/9,042	—	—	—
Rate per 1,000	22.39	19.13	1.19 (0.90–1.57)	1.16 (0.88–1.51)	1.01 (0.78–1.31)

Logistic regression with random effect for cluster; covariates for baseline mean cluster household asset score, baseline cluster proportion of Muslim faith, and baseline cluster mortality rate.

OR, odds ratio.

The findings did not differ substantially when we repeated the analysis to include only women who had lived in the trial clusters for at least a year, when we limited it to the last 2 y of the trial, or when we stratified by socioeconomic status (unpublished data). When we compared a sample of 191 group members with 10,053 non-members in the intervention arm, we found that they had a higher proportion of antenatal care in the public sector (odds ratio 1.52, 95% CI 1.06–2.20), that they were likelier to have rested more in the third trimester of pregnancy (1.74, 1.13–2.67), and that they were likelier to have had a postnatal check-up (1.58, 1.06–2.35). Verbal autopsies were available for 112 stillbirths and 145 neonatal deaths in the 3 y of the trial. The major causes of neonatal mortality were prematurity (40; 27%), intrapartum-related death (33; 23%), infection (33; 23%), and congenital anomalies (10; 7%). There were no obvious differences between allocation groups in the distribution of these causes.

## Discussion

In a cRCT across 48 urban slum communities of Mumbai, it was possible to implement women's groups in challenging conditions. We did not, however, see substantial effects on health care. Women in intervention clusters reported fewer sentinel antenatal morbidities, but we lack a conceptual basis to explain this. The lower stillbirth rate and higher neonatal mortality rate in the intervention arm have, we think, three possible explanations. The first is chance, or residual confounding: it is possible that intervention and control groups differed systematically in ways unaccounted for by our analysis. Second, the intervention may have reduced stillbirths by encouraging timely care-seeking. It is conceivable that some of these infants did not survive subsequently, shifting the balance of mortality from stillbirth to neonatal death. If this was the case, we might have expected to see fewer fresh stillbirths and more early neonatal asphyxial deaths in the intervention arm, a finding not supported by the verbal autopsy data (unpublished data). Third, the data collection may have itself led to improvements in both trial arms. Given that it consisted of birth identification and an interview at 6 wk postpartum, we think this unlikely.

Municipal figures for the same period confirm a reduction in mortality across the city. The positive changes seen in both intervention and control groups are intriguing. We considered the possibility that external initiatives might have affected intervention and control groups, differentially or equally. Significant municipal initiatives in the trial period included some improvement in outreach services by community health volunteers, birth registration and pulse polio campaigns, and infectious disease surveillance. We have cluster-specific information on concurrent non-governmental initiatives. Two NGOs were working on general health in some clusters. We do not think that specific initiatives explain our findings. More likely, in our opinion, was a general improvement in environmental conditions accompanied by behaviour change. Conditions in slum areas improved manifestly over the trial period. Gutters were covered, sanitation block coverage increased, housing fabric became more durable, and there was widespread electricity supply. We think that health-related culture change is a natural accompaniment, all the more because of aspiration and the notions of modernity of Mumbai's residents.

The surveillance and intervention teams were separate. Each of the six municipal wards had a surveillance supervisor and two investigators, responsible for data collection in all eight clusters: a mix of intervention and control. The procedures were identical in intervention and control clusters, and the supervisors and investigators saw their work as unconcerned with the intervention. It is conceivable that in intervention areas *sakhis* could have told identifiers about births and deaths. We have discussed this with the field and data management teams, and we do not think that this happened. As local residents, the birth and death identifiers were aware that there was an intervention in their community, but were focused on their task and did not dwell on the comparative nature of the trial.

The trial demonstrated the value of a counterfactual control group and the potential weakness of ecological evaluation, the commonest example being a before-after comparison. If we had based our assessment on the trends in the intervention arm seen in Figures 3 and 4 (a design used by many programs), it would have appeared an unqualified success [36]. A fall in documented births in the third year of the trial was partly explained by demolition of some settlements, and by difficulties in follow-up. This illustrates a key limitation of urban initiatives: population mobility and the fact that, the poorer the target group, the more transient are their homes. Public health trials would benefit from census data and better registries. Good registration would certainly help when outcome numbers are small and a single missed stillbirth has a substantial effect on a rate per thousand. Our subsequent trial will use censuses rather than prospective ascertainment.

We think that the trial raises three general issues: coverage, target group, and the emergent pattern of health care in cities in the South. Achieving sufficient intervention coverage has been a challenge in other settings [6],[24]. In a situation of space and time constraint, with a lack of social cohesion despite high population density, we did not manage to trigger diffusion of innovation. Using population estimates for Mumbai slum areas from the most recent National Family Health Survey (4.7 members per household; women aged 15–49 constituting 26.7% of the population) [26], our women's groups at their peak involved 8%, and at their nadir 2%, of women of reproductive age (although, as mentioned earlier, outreach communication may have multiplied these figures by up to six times).

Convening community groups was feasible and learning and behaviour change possible, but achieving the impetus necessary for wider change was challenging: group members helped others individually but balked at collective strategizing. There was attrition in group numbers over the course of the intervention, suggesting that women stopped attending when they felt that they had either learned enough or were required to invest more time and energy. Collective action was clearly a big step for groups to take, possible challenges being time-poverty and restrictions on movement, concerns about tenure, lack of confidence, and perhaps a lack of conviction in perinatal health as a major issue. Our target group were slum dwellers, but not exclusively the poorest among them. In a city in which more than half of the population live in slums, slum households themselves encompass a spectrum of socioeconomic realities [16],[37]. For potential replicability, the model included municipal wards with a range of infant mortality rates and, although restricted to slum populations, the intervention may not have succeeded in mobilizing the poorest and most at risk, who tend to be hardest to reach. Although socioeconomic status was not associated with differences in trial outcomes between intervention and control arms, our other research in the same population has demonstrated associations between dimensions of vulnerability and maternal and newborn health risks [38] and we have described inequities in access to routine maternal health services [16],[39] and morbidity care [40]. On the basis of these findings, our strategy has changed: subsequent interventions will target the most vulnerable families and we will intensify our efforts to improve quality of care in private and public facilities.

The third issue was the complexity of urban health care. Antenatal care was the norm and the nadir for institutional delivery in trial clusters was 75%. Around 57% of antenatal care and 30% of deliveries were in the private sector (this in a slum-dwelling population). Open access to private providers, and to institutions at all levels of the public sector hierarchy, is a challenge to systematic health care delivery. Our findings confirmed the tendency to bypass public maternity homes, which should handle uncomplicated deliveries, in favour of tertiary institutions. Women's group discussions included clarification of appropriate sites of consultation and considerations of price and quality, reflected in higher use of public sector services by group members. The study underlined the need to work on quality of care in both public and private sectors. Although regulatory insufficiencies make intervention difficult, quality control in the private sector needs to feature more in debates about health care in low-income countries.

The wider implications of our findings include a tipping of the balance of perinatal intervention in cities toward improvement in service quality, with an emphasis on intrapartum vigilance and resuscitation. Indeed, there have been concerns about the utility of cash incentives for institutional delivery in the urban context, the argument being that access is not the primary issue. Our question was not whether women's groups were beneficial to their members. Members valued the groups and their opportunities for peer learning, showed behaviour change, and helped other women in their communities. Exchange of knowledge about health and health services, rights, social networks, and increased confidence are public goods, although there are challenges in quantifying such outcomes in public health terms. Rather, the question was about the added value of women's groups—over and above activities to improve health care quality—in terms of measurable changes in perinatal health at population level. While acknowledging the possibility that others might be able to achieve this through more intensive community activities in higher mortality settings, our own programme did not show effect. Community groups will feature in our subsequent interventions, as they must in any participatory initiative. We will, however, attempt to integrate them more strongly with pro-poorest targeting, service provision at household level, strengthening of links between communities and service providers, and partnerships with public and private sector providers to improve quality of care.

## Supporting Information

Text S1 **CONSORT checklist.**
(DOC)

Text S2 **Trial protocol.**
(PDF)

Text S3 **Questionnaire.**
(DOC)

## Abbreviations

cRCT: cluster randomised controlled trial
NGO: non-government organisation
