# Supplementary material for: Community Mobilization in Mumbai Slums to Improve Perinatal Care and Outcomes: A Cluster Randomized Controlled Trial
Source: PLoS Med. 2012 Jul 3;9(7):e1001257. doi: 10.1371/journal.pmed.1001257 (PMC3389036; doi:10.1371/journal.pmed.1001257)
Supplement: Text S2 — Trial protocol. (PDF) [file pmed.1001257.s002.pdf]

Trials

BioMed Central

Study protocol

Open Access

Cluster-randomised controlled trial of community mobilisation in Mumbai slums to improve care during pregnancy, delivery, postpartum and for the newborn

Neena Shah More[1], Ujwala Bapat[1], Sushmita Das[1], Sarita Patil[1], Maya Porel[1], Leena Vaidya[1], Bhaveshree Koriya[1], Sarah Barnett[2], Anthony Costello[2], Armida Fernandez[1] and David Osrin*[2]

Address: [1]Society for Nutrition, Education and Health Action (SNEHA), Urban Health Centre, Chota Sion Hospital, 60 Feet Road, Shahunagar, Dharavi, Mumbai 400017, Maharashtra, India and [2]UCL Centre for International Health and Development, Institute of Child Health, 30 Guilford St, London WC1N 1EH, UK

Email: Neena Shah More - cinh.community@gmail.com; Ujwala Bapat - cinh.community@gmail.com; Sushmita Das - cinh.community@gmail.com; Sarita Patil - cinh.community@gmail.com; Maya Porel - cinh.community@gmail.com; Leena Vaidya - cinh.community@gmail.com; Bhaveshree Koriya - cinh.community@gmail.com; Sarah Barnett - s.barnett@ich.ucl.ac.uk; Anthony Costello - a.costello@ich.ucl.ac.uk; Armida Fernandez - drfernandez@snehamumbai.org; David Osrin* - d.osrin@ich.ucl.ac.uk

* Corresponding author

Published: 10 February 2008

Trials 2008, **9**:7 doi:10.1186/1745-6215-9-7

Received: 5 December 2007
Accepted: 10 February 2008

This article is available from: http://www.trialsjournal.com/content/9/1/7

© 2008 More et al; licensee BioMed Central Ltd.
This is an Open Access article distributed under the terms of the Creative Commons Attribution License (http://creativecommons.org/licenses/by/2.0), which permits unrestricted use, distribution, and reproduction in any medium, provided the original work is properly cited.

Abstract

Background: The United Nations Millennium Development Goals look to substantial improvements in child and maternal survival. Morbidity and mortality during pregnancy, delivery and the postnatal period are prime obstacles to achieving these goals. Given the increasing importance of urban health to global prospects, Mumbai's City Initiative for Newborn Health aims to improve maternal and neonatal health in vulnerable urban slum communities, through a combination of health service quality improvement and community participation. The protocol describes a trial of community intervention aimed at improving prevention, care seeking and outcomes.

Objective: To test an intervention that supports local women as facilitators in mobilising communities for better health care. Community women's groups will build an understanding of their potential to improve maternal and infant health, and develop and implement strategies to do so.

Design: Cluster-randomized controlled trial.

Methods: The intervention will employ local community-based female facilitators to convene groups and help them to explore maternal and neonatal health issues. Groups will meet fortnightly through a seven-phase process of sharing experiences, discussion of the issues raised, discovery of potential community strengths, building of a vision for action, design and implementation of community strategies, and evaluation.

The unit of allocation will be an urban slum cluster of 1000–1500 households. 48 clusters have been randomly selected after stratification by ward. 24 clusters have been randomly allocated to receive the community intervention. 24 clusters will act as control groups, but will benefit from health service quality improvement. Indicators of effect will be measured through a surveillance system implemented by the project. Key distal outcome indicators will be neonatal mortality and maternal and neonatal morbidity. Key proximate outcome indicators will be home care practices, uptake of antenatal, delivery and postnatal care, and care for maternal and neonatal illness.

Data will be collected through a vital registration system for births and deaths in the 48 study clusters. Structured interviews with families will be conducted at about 6 weeks after index deliveries. We will also collect both quantitative and qualitative data to support a process evaluation.

Trial registration: Current controlled trials ISRCTN96256793

Background and rationale

Child survival, the Millennium Development Goals, and India

After a decade of relative stagnation, the child survival agenda has been reinvigorated by a series of calls for action and the agreement of a set of United Nations Millennium Development Goals. The fourth and fifth goals are linked explicitly with maternal and child survival targets.[1,2] 11 million children under five die each year, 4 million of them in the neonatal period and 98% in developing countries.[3-5] The neonatal proportion has risen as mortality in later infancy has fallen,[5,6] and is the prime obstruction to attaining the child survival target.

Although many countries are unlikely to achieve the goals, India may yet do so.[7] India's current neonatal mortality rate is 44 per thousand live births and accounts for two-thirds of an infant mortality rate of 68 per thousand.[8] India's National Population Policy calls for a reduction of infant mortality to 30 by 2010, which would require a reduction of neonatal mortality to 20 per thousand.[8] Understanding how policies and programmes can achieve such reductions is a central question, both globally and locally, particularly in view of the poor health status of women and children: about 30% of Indian infants are low birth weight and 45% of children are stunted.[9,10]

The urban future

We will live on a planet of slums.[11] Half the world's poorest residents will be urban by 2035,[12] India's urban poor have outnumbered the rural poor since 1998 and the urban population will reach 610 million by 2025.[13] While the meaning of the word slum remains contentious, the 2001 census of India adopts an inclusive definition, including all areas notified as slums by state or local government and all areas recognized as such even if not formally notified. Slums are described as compact areas with populations of at least 300 (60–70 households), living in poorly built, congested dwellings in an unhygienic environment. Infrastructure, sanitary and drinking water amenities are usually lacking. In 2002, a UN expert group recommended a provisional operational definition based on inadequate access to safe water, inadequate access to sanitation and other infrastructure, poor structural quality of housing, overcrowding, and insecure residential status.[14]

Slum populations rank among the poorest, most underserved and most vulnerable to shocks and health threats.[15] Health is compromised by the often unauthorized status of slums and their poor environmental conditions: housing, sanitation, pollution, water, electricity and health services.[16,17] Associations between such living conditions and illness in mothers and children have been described in South Asian slums.[18-25] In Mumbai slums, three-quarters of women may be anaemic, and low birth weight prevalence may be double the urban average.

Responses to urban health challenges

Because pregnancy and the neonatal period have been invisible over much of the world, recognizing problems, deciding to act on them, reaching a point of care and receiving appropriate care are all problematic. Care for women during pregnancy, and for newborn infants, has not figured explicitly on the agenda of health care providers until recently. In Delhi slums, only 13% of newborn infants with symptoms requiring hospitalisation were referred to hospitals, and when they arrived they were often turned away or managed inappropriately.[23] Although urban India has a relatively strong health and nutrition infrastructure – with public sector investments coming from central, state and local bodies as well as a vast private sector – vulnerable urban communities continue to be poorly served. This is not only the result of under-provision: existing public infrastructure is often sub-optimally used. Rather, it is the product of interrelated factors such as underdevelopment, inequitable distribution of primary healthcare services, poor referral systems, inadequate inter-sectoral linkages, vertical programming, human resource commitment, attitudinal and management challenges, and inefficiency of data management systems.[13] This mesh of influences also includes socioeconomic and cultural determinants such as caring practices, the status of women, the nature of livelihoods, food security, migration and mobility.

Current knowledge of maternity care in Mumbai slums

Information on maternity practices and care-seeking is available from a cross-sectional study undertaken by our group in collaboration with Monitoring and Research Systems, Mumbai. The study interviewed women in Dharavi, familiar to many as Asia's largest slum area, which has seen successive waves of development and community action. 56% of respondents had been married by the age of 18 and 63% had three or more children. 35% of

women slum residents were illiterate and most (91%) did not work outside their cramped homes. Registration of pregnancy was high (95%), but late, and only 18% of women had been visited by a health worker during pregnancy. In case of illness, 52% of women had consulted private practitioners and 34% government services. 35% of women reported eating less than normal during pregnancy and 60% reported no change in the amount of rest they took. Although 91% of women had delivered in a health facility – where attendance by health care professionals was relatively swift and appropriate examination and advice had usually been given – a third had arrived less than an hour before delivery. About one third of infants were recorded as having low birth weight, and breastfeeding was almost universal but not exclusive.

Community interventions for maternal and neonatal survival

Participatory approaches have long been advocated in order to build links between primary services and their users,[26,27] and to improve service quality.[28,29] However, the evidence base for participatory models and their effectiveness remains scanty.[30,31] Didactic presentation of information does not seem to change infant care practices and care seeking behaviour,[32] and some other approach is needed.

The Warmi project, implemented in Bolivia in a poor, rural population of 15,000 with little health system infrastructure, worked with women's groups to encourage participatory planning for mother and infant care,[33] and documented a fall in perinatal mortality from 117 to 44 per thousand births over three years. Although the design lacked power and a control group, it suggested that a participatory approach might have more effect on perinatal care practices and might increase consultation in high risk pregnancies and for at-risk newborn infants.

Building on this example, we conducted the MIRA Makwanpur trial in a population of 170,000 in a mountainous rural district of Nepal. One woman facilitator per population cluster of 7000 facilitated monthly meetings with women's groups to address the issues of pregnancy, childbirth and newborn health. Each group moved through a participatory planning cycle to explore perinatal care strategies and solutions.[34,35] The large cluster randomised controlled trial suggested that participatory work with women's groups could reduce neonatal mortality by 30%.[36]

What is not known

Despite several studies, assumptions and advocacy, the maternal and neonatal situation in slum communities has not been adequately described. Hard indicators – particularly mortality rates – and patterns of care and service usage have been inferred rather than measured. The Municipal Corporation of Greater Mumbai collects vital registration data from hospitals, crematoria and ward offices, and its epidemiological cell is able to derive fairly robust estimates of maternal and infant mortality at city level. Comparisons between areas, particularly between slum and non-slum, are difficult, however, because of the nature of the data.

The particular characteristics of the City Initiative trial in Mumbai are its urban setting, a focus on the poorest groups, an examination of health vulnerability and a multisectoral research partnership. The MIRA Makwanpur trial was conducted in rural Nepal and its external validity requires examination. Key questions include: (1) Will community-based action groups reduce neonatal mortality in urban settings? (2) Is the intervention even feasible in the urban context? (3) Can the intervention model be modified to improve cost-effectiveness? (4) How can government be engaged in research to leverage policy change or adoption? (5) Can community group activities stimulate changes in other outcomes? (6) Can the momentum of change be sustained by community ownership of the process? Amid a wealth of discussion of urbanisation, the project will examine the health of women and children in a hallmark urban context. It represents applied research in a developing country and accords with an agenda to conduct effectiveness studies of interventions, collaborate cross-sectorally, and develop public health research capacity in partner organizations.

Objectives

Goal

To improve the survival and health of mothers and newborn infants in slum communities in Mumbai, India.

Purpose

To test an intervention that mobilises communities for better health care, in which local women's groups build an understanding of their potential to improve maternal and infant health and develop and implement strategies to do so.

Objectives

To test the effect of a participatory intervention using action research cycles with community groups, on:

(a) Care practices and health care seeking behaviour for women in the antenatal, delivery and postpartum periods, and for infants in the neonatal period.

(b) Neonatal and maternal morbidity.

(c) Neonatal mortality.

(d) To document and evaluate the process for potential replicability and sustainability.

Design

The intervention will be evaluated in a cluster-randomised controlled trial. A cluster design has been chosen because the allocation and loci of delivery of the interventions (community clusters) are groups rather than individuals. 48 vulnerable slum clusters have been identified. 24 have been allocated to receive the intervention and 24 will act as controls. An independent surveillance system for outcomes – births, morbidity, mortality, and care seeking – has been designed and implemented in all 48 clusters, covering a population of 350,000.

Research questions

Primary question

Will a community mobilisation intervention improve maternal and neonatal home care, service uptake, morbidity and mortality in slum communities of Mumbai?

Ancillary questions

What are the patterns of maternal and neonatal care in vulnerable slum communities in Mumbai?

What are the neonatal mortality rates in vulnerable slum communities?

How can we assess health vulnerability, how does a cluster-level classification compare with an individual-level one, and is this classification associated with health outcomes?

Hypotheses

Community group activities will lead to: better home care; increased service uptake for routine antenatal, delivery, postnatal and neonatal care; increased care seeking at appropriate facilities for maternal and neonatal illness; reduced neonatal and maternal morbidity; reduced neonatal mortality.

Study endpoints

The study will have three tiers of outcomes (Figure 1).

Tier 1: proximate outcomes at community level. Patterns of routine antenatal, delivery and postnatal care will be described, whether at home or in the informal, public or private sector. In the event of illness, we shall document care at home and care seeking in both private and public sectors.

Tier 2: proximate outcomes at health service level. Patterns of antenatal, delivery and postnatal care will be described in terms of the uptake of public health services. Care seeking for maternal and neonatal illness will be described as it relates to public sector services and facilities.

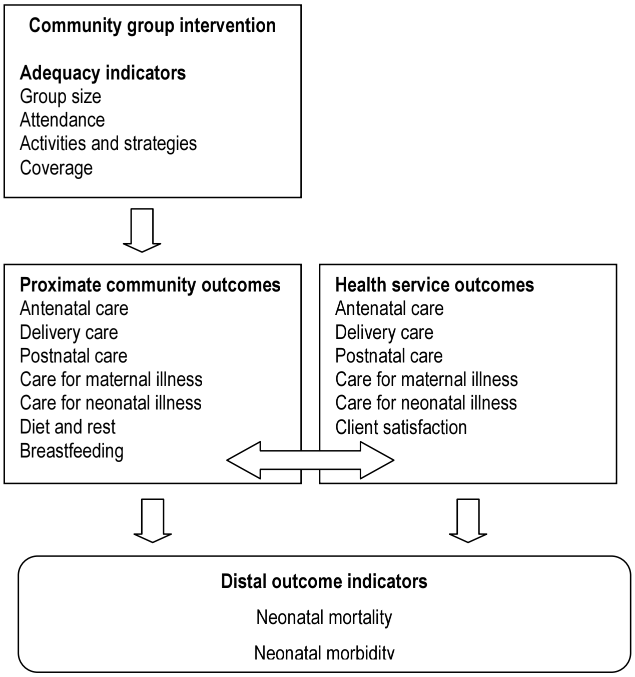

Figure 1
Outcomes of the trial.

Tier 3: distal impacts. Incidence, type and outcome of maternal and neonatal morbidity will be described. Neonatal mortality rates, causes and care seeking will be described. Sample size has been estimated to capture changes in mortality rates in particular.

Methods

Setting

India's most populous city, Mumbai has over 16 million residents and a social heterogeneity which cuts across regional, ethnic, cultural and linguistic lines. The city is divided administratively into 24 municipal wards, within which more than half the population live in slums. The study will be conducted in 6 municipal wards which define the population for a larger cross-sectoral initiative. The City Initiative for Newborn Health is a collaboration between government (the Municipal Corporation of Greater Mumbai, responsible for public health and facilities), a non-government organisation (SNEHA, a non-government organisation committed to maternal and newborn health), an Indian private entity (the Social Initiatives Group of ICICI Bank, India), and an academic research group (the UCL Centre for International Health and Development, UK). The Initiative has two threads, on the demand and supply sides: community mobilisation

activities – the evaluation of which is the subject of the proposal – and improvement of antenatal, delivery and postnatal care at municipal health posts, maternity homes, peripheral hospitals and tertiary hospitals. The initiative is also working on improving the communication and referral procedures between health facilities. The Initiative's approaches are to work with existing systems, to forge partnerships, to build and test replicable models, to focus on current strengths through a participatory approach, to examine the issues of social capital and empowerment in vulnerable groups, and to advocate for policy change.

Target group

Key participants will be women who join community groups. We will try to involve young women and mothers in group activities, but older women may also become involved. Group members will reach out to other women and key stakeholders in their neighbourhoods. Since the aim is to improve the health of pregnant women and their newborn infants, any participant who may influence this situation may be involved. Particular stakeholders may be older women, male community members and leaders, health workers and local opinion formers. The project outcomes will be measured by a surveillance system for births and deaths in 48 intervention and control slum clusters. All women and their newborn infants will be eligible to participate in the data collection exercise, for which enrolment will occur as close to delivery as possible.

Intervention group activities

In each intervention cluster, a facilitator (a local woman, preferably married and with children) will convene community groups to explore maternal and neonatal health issues. Group origin and composition will vary: existing groups who meet to discuss issues such as microfinance, slum improvement, cultural issues and specific agendas, as well as newly convened groups. Although women of childbearing age will be a particular focus of group membership, it is likely that mothers-in-law, men and community leaders will be involved.

Groups will meet fortnightly and move through action research cycles (Figure 2). The model for the process will come from five sources: (a) the experience of the Warmi and Makwanpur programmes,[33,36] as well as ongoing work in rural Jharkhand and Orissa and Bangladesh. (b) self-efficacy and empowerment agendas with their roots in social mobilisation movements[37,38], (c) a vision building process informed by appreciative inquiry,[39,40] (d) piloting in non-study areas, and (e) the experience of the facilitators. The group agenda will move through seven phases.

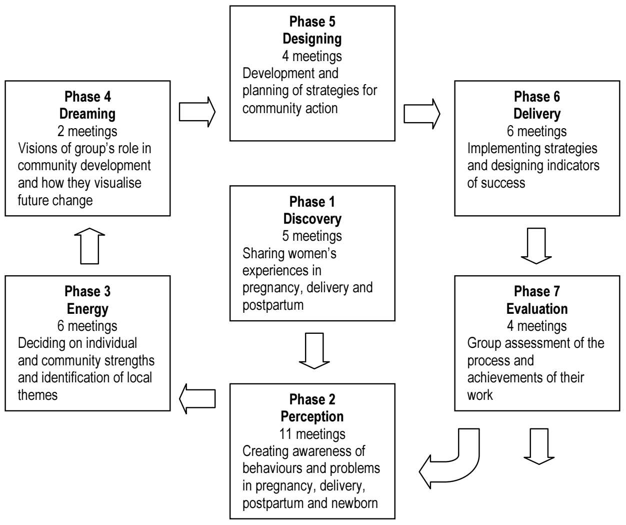

Meeting numbers are rough advance estimates

Figure 2
The sequence of group meetings that constitute the intervention.

In the first phase, group members will discuss the experience of pregnancy, childbirth and infant care in their own communities. We will take a particular interest in local practices. They will ventilate and bring into the group space the rich variety of their individual experiences in varied settings. This will be the first step to peer sharing and learning. In the second phase, picture cards will be used to build perceptions of members on all possible problems and illnesses encountered during maternity and the neonatal period. The discussion will stimulate awareness of local names, symptoms, contributory factors, prevention, care practices and possible treatment. The third phase involves a discovery of the strengths of individual members, their families and communities so as to build the energy for designing and implementing experience-based strategies. In previous programmes we have recommended a problem-focused approach to this, but the intervention will take an appreciative approach based on the vision of a positive experience. In this it has something in common with positive deviance approaches, and will lead to identification of the particular local health issues and challenges. In the fourth phase, group members will construct a dream vision for their slum communities, with an emphasis on maternal and child health. This may involve wider community meetings and efforts to build community commitment and ownership. The vision will in turn lead to design of strategies in the fifth phase, their implementation in the sixth phase and finally their evaluation – by groups themselves – in the seventh phase. Once a primary cycle of meetings has been completed, part of it

may be repeated with the larger community group, involving multiple stakeholders and building the sustainability of the process and ownership of outcomes. Work with the original groups may continue, and new cycles may be set up with other groups.

The programme inputs can be itemised as: (1) recruitment, training, supervision and remuneration of facilitators. The role of the facilitator is to activate and strengthen groups, support them in identifying strengths and focal themes for intervention based on past experiences and collective wisdom, help to plan possible strategies and support their implementation and monitoring in the community. Although she requires a grasp of health issues and some knowledge of potential interventions, she needs to be a facilitator rather than a teacher. As such, she may act as a broker of information and communication but her prime importance is as a catalyst for community mobilisation; (2) development of tools and monitoring mechanisms for conducting group meetings, process evaluation and documentation; (3) recruitment, training, supervision and remuneration of a supervisory cadre to support the community-based facilitators; (4) knowledge and communication with other actors such as local non-government organisations and allied sectoral services; (5) collection of cost data to allow for economic evaluation of the model.

Randomisation and allocation

The sampling frame for the trial contains the most vulnerable areas of slums in the 6 urban wards covered by the City Initiative for Newborn Health (F/N, G/N, H/E, K/W, M/E, P/N). These 6 wards were selected purposively for accessibility to the implementing partners and to reflect a range of infant mortality rates according to Municipal Corporation estimates. Criteria for health vulnerability were developed from discussion with key informants and the literature. These included likelihood of demolition and migration, health service access and utilization, socioeconomic factors (housing ownership and tenure, structure, special groups in difficult circumstances), and environmental factors (sanitation, drainage, water supply, electricity supply). In a two-stage process (Figure 3), the 6 wards of the sampling frame were visited to identify and select health posts and vulnerable slum localities, the criteria were refined, and the localities were visited systematically. For study purposes, the smallest permissible cluster contains 1000 households. 92 candidate vulnerable slum clusters were identified across the 6 wards and 24 health posts involved in the City Initiative for Newborn Health. These clusters constitute the sampling frame.

For equity across the wider initiative, randomisation was stratified by municipal ward. Eight slum clusters in the catchment areas of each of the 24 health posts involved in the City Initiative for Newborn Health were selected randomly from each of the 6 wards in the sampling frame, giving a total of 48 clusters. Four clusters per ward were allocated randomly to the intervention group, and four to the control group. Allocation was not concealed because of the nature of the intervention. However, evaluation teams and intervention teams conduct their activities

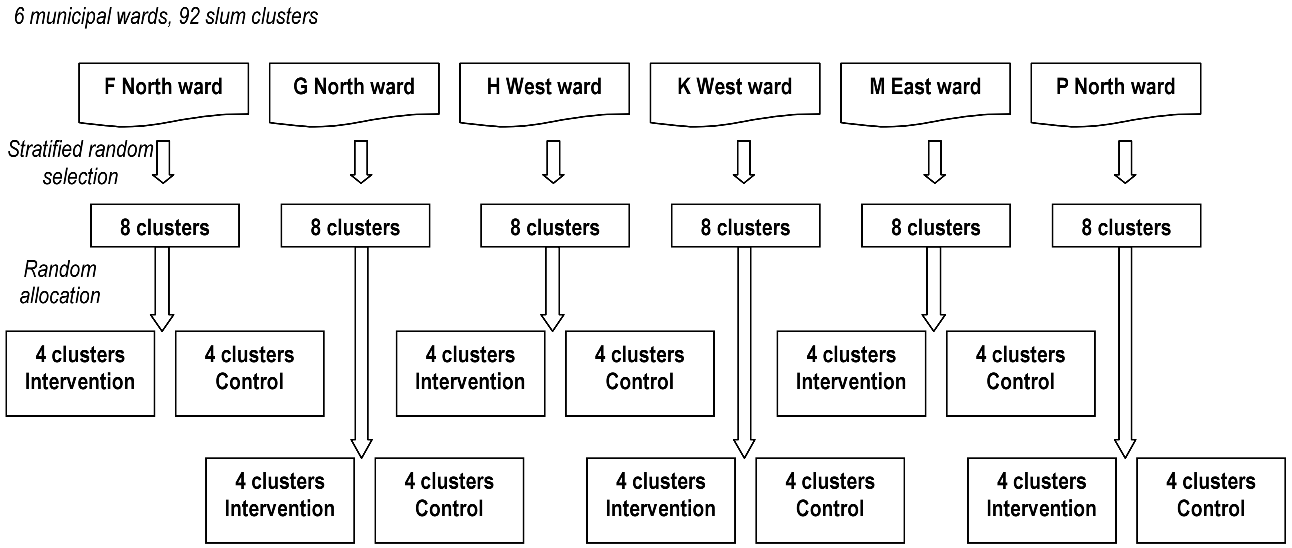

Figure 3
Trial design.

independently and no primary outcome results will be fed back to inform the intervention.

Sample size

We aim to accumulate data on 300 births per cluster over 3 years, which implies a cluster size of 900–1400 households on the basis of municipal demographic data. The surveillance of births and birth outcomes will be carried out in 48 clusters of 1000–1500 households. Sample size was predicated on the primary outcome of neonatal mortality rate. Calculations were based on the equations of Hayes and Bennett,[41] assuming two treatment groups, unmatched clusters of approximately equal size, a value of k – the between-cluster coefficient of variation – equal in intervention and control groups, and the addition of 2 to the estimated cluster number to account for loss of degrees of freedom consequent on stratification. The value of k was set at 0.3 on the basis of (a) available estimates of infant mortality rate in 24 wards of Mumbai from 2003 municipal data, and (b) estimates of neonatal mortality rate from the Makwanpur study in Nepal. Sample size was estimated for a range of births per cluster (200–350) and a range of possible reductions in neonatal mortality rate. At 80% power and two-tailed 5% significance level, the sample size will allow us to detect a reduction in neonatal mortality rate from 30 to 20 per thousand live births. We are aware of a secular trend to decreasing mortality in urban India, and have provisionally analysed vital events for the inception year of the project. If neonatal mortality rates are lower than suggested by the available figures, the sample size will allow us to detect a fall from 20 to 12 per thousand live births at 80% power. Both estimates project significance for a fall of roughly a third in neonatal mortality rate.

Data collection

An independent project vital registration system has been set up in the 48 study clusters. The system is a modification of one that was successful in the Makwanpur study, the modifications being based on experiences of running less intensive systems in Bangladesh, Jharkhand and Orissa. All births, female deaths, stillbirths, neonatal and infant deaths will be monitored by local resident enumerators. These enumerators will communicate with interviewers, who will confirm each event and visit women and their families in their homes to deliver a postnatal interview at about 6 weeks after delivery. The interview will describe the respondent's demographic background, socioeconomic status and maternity history. Subsequent sections will cover antenatal, delivery, postnatal and neonatal care, as well as responses to illness in mother or infant. In the event of maternal death, stillbirth or neonatal death, supervisory staff will collect verbal autopsy information from family members and triangulate it with information available from health care facility documents.

Data management

Hard copy questionnaires are transferred to the central office weekly. Two types of data are entered into electronic relational database management systems in Microsoft Access. Births and deaths are entered into a database dedicated to tracking follow-up and generating mortality rate outcomes. Interview questionnaires are entered into a separate database. Both databases include validation constraints and enforce referential integrity. All information provided by participants remains confidential. Access is restricted to interviewers, supervisors, data auditors, and officers, and research staff at the analytical level. No analyses or outputs include the names of participants.

Quality control

The trial is headed by a project coordinator. Data collection activities are managed by 2 project officers, each responsible for 3 urban wards. 99 female residents of the project clusters are tasked with identifying births and deaths in their areas. Each event is crosschecked by a female cluster interviewer responsible for 4 clusters, who visits the home to verify it and to discuss a follow-up visit with family members. This process is quality controlled by a further visit by a female supervisor responsible for 8 clusters (1 ward) to confirm every fifth event. When interviews are completed, each is checked by the interviewer in the field and by a supervisor. Supervisors also visit homes to crosscheck the data from every 10th interview and to ensure valid responses to queries arising from crosschecking, and make random visits to observe interviews. Every third interview tool is crosschecked by a project officer. Supervisors and officers meet weekly to review progress and issues arising. All questionnaires and memoranda are crosschecked at the central office. Data entry includes internal validation rules. Entered data are internally crosschecked by the data management officer for every 10th questionnaire, and externally crosschecked against every 15th questionnaire.

Dealing with loss-to-follow-up

Although all trials have to deal with loss-to-follow-up, slum communities in Mumbai have some special features. Our estimates suggest that some areas experience 25% annual in- and out-migration. Further, many residents retain connections with their family homes in other parts of India. A number of specific problems arise for birth outcome tracking: (a) residents of other places may come to the city for delivery; (b) residents of slum areas may go to their family homes for delivery (within or outside Mumbai), or for a variable period shortly after it; and (c) instability of tenure and livelihood may contribute to mobility within and outside slum areas; (d) working

women may not be available for interview; (e) demolition of parts of or whole slum areas as part of urban infrastructure initiatives. We are attempting to deal with these issues in the trial, by three main methods. First, we have designed the household surveillance in order to achieve 100 annual births per cluster after potential loss-to-follow-up of 25%. Second, interview times will be flexible and multiple visits have been planned for. Third, the parallel database systems optimise the available information: for a large number of deliveries, we are able to ascertain the outcome at one month postpartum, even if the interviewers do not succeed in completing an interview. This allows us to generate mortality rates from a larger tranche of data, while using interview data from a smaller tranche for the secondary outcomes.

Interim analyses and stopping rules

There will be two types of interim analysis. (1) An *analysis of baseline findings*, particularly neonatal mortality and care-seeking practices. The analysis will not look at outcomes in terms of allocation. The results will provide us with estimates of neonatal mortality rates on the basis of which cluster size will be reviewed. (2) An *interim analysis of outcomes*. This will be conducted in late 2008. The analysis will look at the outcomes in terms of allocation. At this point a Data Safety Monitoring Board (DMB) will be convened according to the DAMOCLES statement.[42] The DMB will examine the study for deviations from protocol and preliminary results. It is unlikely that a community mobilisation intervention will have adverse effects. From our experience in other, similar studies, we do not intend to institute stopping rules. However, if there are social or political problems in specific clusters, each situation will be judged on its merits and the trial can be stopped if necessary. The DMB will also advise on whether the trial should be extended to achieve greater power.

Analysis strategies

The analysis will be undertaken as intention-to-treat at both cluster and participant levels. Participants who begin the trial as residents of a given cluster will be retained as residents even if they move to another cluster during the trial period. Within the prospective cohort, stillbirth and neonatal mortality rates will be compared in control and intervention groups, taking account of clustering, with hierarchical logistic models. Intraclass correlation coefficients will be estimated from preliminary surveillance of neonatal mortality and stillbirth data by analysis of variance. Secondary outcomes and process indicators will also be compared with adjustment for clustering. All estimates will be presented with 95% confidence intervals.

Process evaluation

Alongside the impact evaluation that determines the trial design, we will carry out process evaluation activities. The objectives will be to describe the social context in which the intervention was implemented, what was planned, what was actually delivered, what participants received and how they responded. We will cover planned programme inputs and their iterative development through dialogue with project personnel and community participants. We are particularly interested in the activities of the community-based facilitators: who they are, to what degree they represent their areas, how they respond to their training and work, how able they are to create and sustain viable groups and what they think of the intervention. We will take a similar approach to groups themselves: their size, membership and representativeness, their progress through the groupwork process, their understanding and interest in group activities, and the degree of behaviour change in both group members and the wider community. The spread of knowledge, strategies and behaviour change are of particular interest, and we will explore the possibilities for social network mapping and qualitative research on communication. We will put in place a data collection system to document group attendance, demographics and progress through the intervention cycle. Alongside this – predominantly quantitative – approach, we will conduct qualitative research; the main methods of which will be focus group discussions and semi-structured interviews with project officers, facilitators and community group members.

Economic evaluation

We will conduct an economic evaluation along the lines of the cost-effectiveness work that we have previously published.[43] Trial expenditure is entered into accountancy software (Tally 7.2, Tally Solutions Pvt Ltd, India) under cost codes. We shall evaluate cost-effectiveness in terms of life years saved (LYS), with appropriate discounting.

Ethical issues

Approvals

The trial has been approved by the Municipal Corporation of Greater Mumbai, the Independent Ethics Committee for Research on Human Subjects (Mumbai committee, reference IEC/06/31), and the ethics committee of the Institute of Child Health and Great Ormond Street Hospital for Children. It is registered with ISRCTN96256793 and appears on the Wellcome Trust register, hosted in the meta-Register of Controlled Trials (reference 081052).

Consent

After a full explanation of the data collection activities, participants will be asked for verbal consent to interview and assured of the confidentiality of data. Data will be treated anonymously. All community-based members of the study team will be recruited locally. Team members who encounter illness in mothers or infants will facilitate

attendance at appropriate health facilities, and in cases of severe illness will have an ethical responsibility to recommend and advise on attendance at an appropriate health facility, irrespective of allocation. Group members will be advised that their attendance depends entirely on their own wishes, and that they may choose to attend or leave at any time.

Benefits to control areas

We have equipoise on the intervention under test. Community group activities as a means of addressing maternal and newborn survival have been insufficiently tested for us to have an opinion as to whether they may be successful in the urban context. Since the trial is part of the City Initiative for Newborn Health, control areas will benefit from the initiative's other activities: improvement of provision and quality of maternity and newborn services at health posts, maternity homes, peripheral hospitals and tertiary hospitals. The sampling frame for the trial has been designed to maximise the benefits to communities of supply side activities. Residents of the urban slum clusters involved in the trial fall under the catchment areas of municipal health care facilities involved in these activities.

Sustainability and scalability

The basic framework of the phases and the choice of appreciative inquiry as the primary approach and guiding philosophy were finalized after a trial run of the intervention in a slum area not covered by the project. The detailing of the content for each meeting within each phase of the model will be developed iteratively with the ongoing participation of the facilitators in their weekly meetings. The model is necessarily evolving and this is the foundation for its sustainability. We hope that the methodology ensures that the processes are internalised by the local facilitators and can be replicated to focus on issues other than newborn care. We also hope that outreach by group members to other women and stakeholders in their immediate neighbourhoods build ownership and sustainability for the initiative. The 8–10 women's groups in each cluster may choose to integrate into a large community group and act as representatives for the entire community. This may further evolve into a slum health committee that takes over part remuneration for the facilitator, individual women in the slum paying her services. Community support systems may take time to develop given the existing low levels of social networking. The primary health care centres run by the Municipal Corporation will be responding to the introduction of the national Urban Reproductive and Child Health Programme, and if the intervention is successful we will advocate the assignation of the facilitator role to the community health volunteers it employs. Local non-government organisations focusing on health may also be persuaded to review their present activities and assimilate the model into their agendas if impact is shown. Necessarily and ethically, control areas will be the first candidates for scaling-up.

Public engagement

The project will follow the Towards4+5 research programme consortium communication policy (available on request). We will review the literature on urban maternal and neonatal health care and slums. We will link dissemination with implementation by embedding the project in the public health care system and slum communities, identify potential users in civil society and the public and private sectors, and consider which issues are of interest to them and the type of evidence they prefer. Findings will be disseminated at community level, and the processes of data collection and handling may be opened to community ownership and management at a later date.

We will use a range of copy platforms: a user-friendly protocol, articles in mass media, websites, vernacular summaries in English, Hindi and Marathi. We will exploit opportunities for discussion, particularly public network meetings, academic conferences and local events onto which dissemination can be piggy-backed. We will seek to institutionalise learnings through policy changes, through dialogue with municipal policymakers. We will discuss methodology and findings with researchers and educationists at Indian institutes of excellence, and place and supervise Indian postgraduate students within the project. The results of the trial will be published in an open access academic journal. Summaries will be prepared for dissemination to lay readers and policy makers.

Project administration

SNEHA was founded in 1999 as a voluntary, secular, non-profit organization registered under the Bombay Trust Act of 1950. Its mandate is to work to improve the lives of women and children in urban slum settings, with a specific focus on health and nutrition. Team members have a range of clinical, social work and administrative backgrounds, with an emphasis on equal opportunities and local recruitment. SNEHA has collaborated with the UCL Centre for International Health and Development since 2003, as a result of which research activities and capacity have been consolidated. Responsibility for implementation of the trial rests with SNEHA. The administrative structure is shown in Figure 4. SNEHA currently administers 14 projects and has dedicated management and administrative teams. We have in-house capabilities in human resources management, logistics and accounting.

Trial steering committee

A trial steering committee meets annually to review the conduct and progress of all City Initiative activities. The committee includes representatives from the All India Institute of Medical Sciences, the national Urban Health

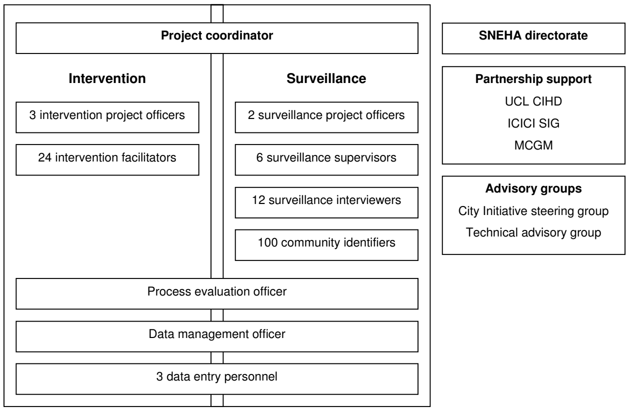

Figure 4
Administrative structure.

Resource Centre, UNICEF, the Tata Institute for Social Sciences, the Municipal Corporation of Greater Mumbai, UCL Centre for International Health and Development, and the ICICI Social Initiatives Group.

Competing interests
The author(s) declare that they have no competing interests.

Authors' contributions
NSM is the project coordinator, contributed to the design of the study, will be responsible for the management of the trial, and will participate in the analysis and interpretation of data. UB, SD and BK contributed to the design of the study, will be responsible for the collection and management of surveillance data, and will participate in their analysis and interpretation. SP, MP and LV contributed to the design of the intervention, will be responsible for its implementation, and will participate in the analysis and interpretation of data. AF is the director of SNEHA, contributed to the design of the study, and will have overall responsibility for the trial in Mumbai. AC is the director of the UCL Centre for International Health and Development, contributed to the design of the study, and will have overall responsibility for UK partner contributions. SB contributed to the design of the study and the process evaluation, and will participate in the analysis and interpretation of data. DO wrote the first draft of the protocol and, with NSM, coordinated its development and gave final approval for the version to be published. AC, AF, SB, NSM and DO obtained grant funding. All authors contributed to critique and modification of the manuscript.

Acknowledgements
The trial is part of the City Initiative for Newborn Health, funding for which has been provided by ICICI Bank Social Initiatives Group. Representatives of this group are involved in periodic discussions of the role of the trial in Indian public health research, and in generating ideas for areas of study. From early 2007, part funding for research and evaluation activities has been provided by The Wellcome Trust as part of a career development fellowship for DO. Neither funding body had a role in study design, the collection, analysis or interpretation of data, the writing of the manuscript, or the decision to submit it for publication.

We thank Roopashri Sinha and Glyn Alcock for work on the process evaluation, Gayatri Raman and Kshipra Tendolkar for work on developing the intervention, Jaya Nuty and Wasundhara Kanbur for contributions to the development of the intervention and for facilitator training inputs, Sushma Shende for inputs on primary health care provision, and Shilpa Deshpande, Devanshi Chanchani, Mekhala Krishnamurthy and Abhijit Visaria for ongoing support and discussions of the study.

References
1. UN General Assembly 56th session: **Road map towards the implementation of the United Nations millennium declaration: report of the Secretary-General (UN document no. A/56/326).** New York: United Nations; 2001.
2. **Millennium Development Goals** [http://www.un.org/millenniumgoals]. Accessed 10/07/2004
3. WHO: **Perinatal mortality: a listing of available information.** Geneva: World Health Organization, FRH/MSM/96.7; 1996.
4. Saving Newborn Lives: **State of the world's newborns.** Washington DC: Save the Children; 2001.
5. Black R, Morris S, Bryce J: **Child survival I. Where and why are 10 million children dying every year?** *Lancet* 2003, **361:**2226-2234.
6. Claeson M, Bos E, Mawji T, Pathmanathan I: **Reducing child mortality in India in the new millennium.** *Bull World Health Organ* 2000, **78:**1192-1199.
7. World Bank: **Global economic prospects and the developing countries 2002: making trade work for the world's poor.** Washington, DC: World Bank; 2002.
8. NNF: **The State of India's Newborns.** New Delhi & Washington DC: National Neonatology Forum & Save The Children US; 2004.
9. UNICEF: **The state of the world's children 2006: excluded and invisible.** New York: United Nations Children's Fund; 2005.
10. IIPS: **National Family and Health Survey II, 1998–1999.** Mumbai: Indian Institute of Population Studies; 2000.
11. Davis M: **Planet of slums.** London & New York: Verso; 2006.
12. Ravallion M: **On the urbanization of poverty.** In *Policy research working paper 2586* Washington DC: World Bank; 2001.
13. EHP: **Report on national consultation on improving the health of the urban poor. Bangalore, 30th June – 1st July 2003.** Bangalore: Environmental Health Project; 2003.
14. United Nations Human Settlements Programme (UN-Habitat): **The challenge of slums: global report on human settlements 2003.** London and Sterling VA: Earthscan Publications Ltd; 2003.
15. World Bank: **Report of a consultation on the health of the poor in urban India.** New Delhi: World Bank; 2002.
16. Gopalan C: **The urban challenge – health and nutrition implications.** *NFI Bull* 1992:13.
17. Ghosh S, Shah D: **Nutritional problems in urban slum children. Environmental Health Project, special article series.** *Ind Pediatr* 2004:41.
18. Bhargava S, Singh K, Saxena B: **ICMR Task Force National Collaborative Study on identification of high risk families, mothers and outcome of their off-springs with particular reference to the problem of maternal nutrition, low birth weight, perinatal and infant morbidity and mortality in rural and urban slum communities.** *Ind Pediatr* 1991, **28:**1473-1480.
19. Awasthi S, Pande V, Glick H: **Under fives mortality in the urban slums of Lucknow.** *Ind J Pediatr* 1996, **63(3):**363-368.
20. Kapoor R, Srivastava A, Misra P, Sharma B, Thakur S, Srivastava K, Singh G: **Perinatal mortality in urban slums in Lucknow.** *Ind Pediatr* 1996, **33(1):**19-23.
21. Hussain A, Ali S, Kvale G: **Determinants of mortality among children in the urban slums of Dhaka city, Bangladesh.** *Trop Med Int Health* 1999, **4:**758-764.
22. D'Souza R, Bryant J: **Determinants of childhood mortality in slums of Karachi, Pakistan.** *J Health Popul Developing Countries* 1999, **2(1):**33-44.

23. Bhandari N, Bahl R, Taneja S, Martines J, Bhan M: **Pathways to infant mortality in urban slums of Delhi, India: implications for improving the quality of community- and hospital-based programmes.** *J Health Popul Nutr* 2002, **20:**148-155.
24. Fernandez A, Mondkar J, Mathai S: **Urban slum-specific issues in neonatal survival.** *Ind Pediatr* 2003, **40(12):**1161-1166.
25. Awasthi S, Agarwal S: **Determinants of childhood mortality and morbidity in urban slums in India.** *Ind Pediatr* 2003, **40(12):**1145-1161.
26. Stone L: **Primary health care for whom? Village perspectives from Nepal.** *Soc Sci Med* 1986, **22:**293-302.
27. Justice J: **Neglect of cultural knowledge in health planning: Nepal's Assistant Nurse-Midwife program.** In *Anthropology and public health: bridging differences in culture and society* Edited by: Hahn R. New York: Oxford University Press; 1999.
28. Kutzin J: **Obstacles to women's access: issues and options for more effective interventions to improve women's health.** In *Human Resource Development and Operations Policy (HRO) Working Paper no. 13* Washington DC: World Bank; 1993.
29. Tipping G, Segall M: **Health care seeking behaviour in developing countries – an annotated bibliography and literature review.** In *Development Bibliography 12* Sussex: Institute of Development Studies; 1995.
30. Morgan L: **Community participation in health: perpetual allure, persistent challenge.** *Health Policy Plan* 2001, **16:**221-230.
31. Ensor T, Cooper S: **Overcoming barriers to health service access: influencing the demand side.** *Health Policy Plan* 2004, **19:**69-79.
32. Bolam A, Manandhar D, Shrestha P, Malla K, Ellis M, Costello AM: **The effects of postnatal health education for mothers on infant care and family planning practices in Nepal: a randomised, controlled trial.** *Br Med J* 1998, **316(7134):**805-811.
33. Howard-Grabman L, Seoane G, Davenport C, MotherCare and Save the Children: **The Warmi Project: a participatory approach to improve maternal and neonatal health, an implementor's manual.** Westport: John Snow International, Mothercare Project, Save the Children; 2002.
34. Osrin D, Mesko N, Shrestha B, Shrestha D, Tamang S, Thapa S, Tumbahangphe K, Shrestha J, Manandhar M, Manandhar D, *et al.*: **Implementing a community-based participatory intervention to improve essential newborn care in rural Nepal.** *Trans Roy Soc Trop Med Hyg* 2003, **97:**1-4.
35. Morrison J, Tamang S, Mesko N, Osrin D, Shrestha B, Manandhar M, Manandhar D, Standing H, Costello A: **Women's health groups to improve perinatal care in rural Nepal.** *BMC Pregnancy and Childbirth* 2005, **5(1):**6.
36. Manandhar D, Osrin D, Shrestha B, Mesko N, Morrison J, Tumbahangphe K, Tamang S, Thapa S, Shrestha D, Thapa B, *et al.*: **Effect of a participatory intervention with women's groups on birth outcomes in Nepal: cluster randomized controlled trial.** *Lancet* 2004, **364:**970-979.
37. Bandura A: **Self-efficacy: toward a unifying theory of behavioural change.** *Psychological Rev* 1977, **84(2):**191-215.
38. Freire P: **Pedagogy of hope: reliving pedagogy of the oppressed.** New York: Continuum International Publishing Group; 1995.
39. Trosten-Bloom A, Whitney D: **Appreciative Inquiry: the path to positive change.** In *Managing change in healthcare: innovative solutions for people-based organizations* Edited by: Key M. McGraw-Hill Companies; 1999:113-128. [Hfma Healthcare Financial Management Series].
40. Pradhan R: **The transformative power of appreciative conversations: a case study in hospitals in South Asia.** *AI Practitioner* 2004, **24:**19-24.
41. Hayes R, Bennett S: **Simple sample size calculation for cluster-randomized trials.** *Int J Epidemiol* 1999, **28:**319-326.
42. DAMOCLES Study Group: **A proposed charter for clinical trial data monitoring committees: helping them to do their job well.** *Lancet* 2005, **365:**711-722.
43. Borghi J, Thapa B, Osrin D, Jan S, Morrison J, Tamang S, Shrestha B, Wade A, Manandhar D, Costello A: **Economic assessment of a women's group intervention to improve birth outcomes in rural Nepal.** *Lancet* 2005, **366:**1882-1884.

Publish with **BioMed Central** and every scientist can read your work free of charge

"BioMed Central will be the most significant development for disseminating the results of biomedical research in our lifetime."

Sir Paul Nurse, Cancer Research UK

Your research papers will be:

- available free of charge to the entire biomedical community
- peer reviewed and published immediately upon acceptance
- cited in PubMed and archived on PubMed Central
- yours — you keep the copyright

Submit your manuscript here:
http://www.biomedcentral.com/info/publishing_adv.asp

BioMedcentral
