# Supplementary material for: Community Mobilization in Mumbai Slums to Improve Perinatal Care and Outcomes: A Cluster Randomized Controlled Trial
Source: PLoS Med. 2012 Jul 3;9(7):e1001257. doi: 10.1371/journal.pmed.1001257 (PMC3389036; doi:10.1371/journal.pmed.1001257)
Supplement: Text S3 — Questionnaire. (DOC) [file pmed.1001257.s003.doc]

***TRANSLATED FROM HINDI***

Interview start time ⬜⬜⬜⬜

**SNEHA**

**Basti level birth surveillance questionnaire**

**City Initiative for Newborn Health**

***This questionnaire should be used only if the baby is alive***

| Survival status of mother | | Alive ⬜ | | Died ⬜ |
| --- | --- | --- | --- | --- |
| Who were present during the interview besides the respondent? | No one ⬜ | Husband ⬜ | In-laws ⬜ | Any other ⬜ |

***If the mother has died, ask this questionnaire to family members who are best able to answer.***

***Before the interview, request the mother or family members to collect all medical records, antenatal care records, delivery records and any others relating to mother and baby.***

|  | **Section A: Administrative** | | | | | | | | | | | | | | | | | | | | | | | | | | | | | | |
| --- | --- | --- | --- | --- | --- | --- | --- | --- | --- | --- | --- | --- | --- | --- | --- | --- | --- | --- | --- | --- | --- | --- | --- | --- | --- | --- | --- | --- | --- | --- | --- |
| A1 | Ward | | | | F/N ⬜ | | | | | G/N ⬜ | | | | | | | | | H/E ⬜ | | | | K/W ⬜ | | | | | M/E ⬜ | | | P/N ⬜ |
| A2 | Cluster number | | ⬜⬜ | | | | Mother number | | | | | | | | | | | ⬜⬜⬜ | | | | | | Baby number | | | | | ⬜⬜⬜ | | |
| A3 | Household address | | | | | | | ………………………………………………………………………… | | | | | | | | | | | | | | | | | | | | | | | |
| A4 | Name of respondent | | | | | | | ………………………………………………………………………… | | | | | | | | | | | | | | | | | | | | | | | |
| A5 | Consent section | | | | | | | | | | | | | | | | | | | | | | | | | | | | | | |
|  | *Translate consent section* | | | | | | | | | | | | | | | | | | | | | | | | | | | | | | |
| A6 | Number of visits made to complete interview | | | | | | | | | | | | | | | | | | | | | ⬜ | | | | | | | | | |
|  | If more than 1 visit, reason …………………………………………………………….. | | | | | | | | | | | | | | | | | | | | | | | | | | | | | | |
| A7 | Language of interview | Translator (**specify language**) ⬜ ………………… | | | | | | | | | | | | | | | | Hindi ⬜ | | | Marathi ⬜ | | | | | | Gujarati ⬜ | | | | English ⬜ |
| A8 | Date of interview | | | _ _ / _ _ / _ _ _ _ *day/month/year* | | | | | | | | | | | | | | | | | | | | |  | | | | | | |
|  |  | | | | | | | | | | | | | | | | | | | | | | | | | | | | | | |
|  | *Interviewer check* | | | | | | | | | | | | | | | | | | | | | | | | | | | | | | |
| A9 | Questionnaire complete | | | | | | | | | | | | | | Yes ⬜ | | | | | | | No ⬜ | | | | | | | | | |
| A10 | Reason for incompleteness | | | | | | | | …………………………………………………………………………………………………….………….………….………….……… | | | | | | | | | | | | | | | | | | | | | | |
|  | Interviewer signature ……………………… | | | | | | | | | | | | | Write full name ………………………………………. | | | | | | | | | | | | | | | | | |
|  | *Supervisor check* | | | | | | | | | | | | | | | | | | | | | | | | | | | | | | |
| A11 | Date of check | | | | | _ _ / _ _ / _ _ _ _ *day/month/year* | | | | | | | | | | | | | | | | | | | | | | | | | |
|  | Supervisor signature ……………………… | | | | | | | | | | | | | Write full name ………………………………………. | | | | | | | | | | | | | | | | | |
| A12 | Date of receipt at office | | | | | _ _ / _ _ / _ _ _ _ *day/month/year* | | | | | | | | | | | | | | | | | | | | | | | | | |
| A13 | Location of woman | | | | | Lives in basti ⬜ | | | | | | | | | | | Just arrived in basti ⬜ | | | | | | | | | Moved out of basti ⬜ | | | | | |
| A14 | How long have you been living in this basti? | | | | | | | | | | | | ⬜⬜ *months* or ⬜⬜ *years* | | | | | | | | | | | | | | | | | All her life ⬜ | |
|  | ***If less than 1 month, write 01 in months*** | | | | | | | | | | | | ***If over 1 year go to A16*** | | | | | | | | | | | | | | | | |  | |
| A15 | Did you come here just for the pregnancy/delivery? | | | | | | | | | | | | | | | | | | | | | | | | | | | | | | |
|  | Other ⬜ | | | | | | | | | | Family moved to this basti not linked to pregnancy ⬜  Came for pregnancy/delivery and intend to return home ⬜  Came for pregnancy/delivery but now intend to stay ⬜ (specify) …………………………………………………… | | | | | | | | | | | | | | | | | | | | |
| A16 | From your current pregnancy till date did you moved away from this residence for more than 40 days? | | | | | | | | | | | | | | | | | | | | | | | | | | | | | | |
|  | Yes ⬜ | | | | | | | | | | | No ⬜ | | | | | | | | How long ⬜⬜ days ⬜⬜ months | | | | | | | | | | | |
| A17 | Do you have the antenatal case paper? | | | | | | | | | | | | | | | Yes ⬜ | | | | | | | | | | | | | | No ⬜ | |

|  | **Section B: Background characteristics** | | | | | | | | | | | | | | | | | | | | | | | | | | |
| --- | --- | --- | --- | --- | --- | --- | --- | --- | --- | --- | --- | --- | --- | --- | --- | --- | --- | --- | --- | --- | --- | --- | --- | --- | --- | --- | --- |
| B1 | How old were you on your last birthday? ***(Completed years)*** | | | | | | | | | | | | | | | ⬜⬜ *years* | | | | | | | | | | Don’t know ⬜ | |
| B2 | How old were you when you first got married? | | | | | | | | | ⬜⬜ *years* | | | | | | Not married ⬜ | | | | | | | | | | Don’t know ⬜ | |
| B3 | How old were you when you first became pregnant? | | | | | | | | | | | | | | | ⬜⬜ *years* | | | | | | | | | | Don’t know ⬜ | |
| B4 | Did you take infertility treatment for this pregnancy? | | | | | | | | | | | | | | | Yes ⬜ | | | | | | | | | | No ⬜ | |
| B5 | What sort of education have you had? | | | | | | | No education at all ⬜ | | | | | | | | | | | | Informal education ⬜ | | | | | | | |
|  |  | School up to class ⬜⬜ | | | | | | | | | | | | | | | | | | | | | College ⬜⬜ | | | | |
| B6 | Can you read? | | Yes ⬜ | | | | No ⬜ | | | | | | |  | | | | | | | | | | | | | |
| B7 | What is your religion? | | Hindu ⬜  Muslim ⬜ | | | | Christian ⬜  Sikh ⬜ | | | | | | | Buddhist ⬜  Jain ⬜ | | | | | | | Parsi ⬜  No religion ⬜ | | | | | | |
|  |  | | Other ⬜ | | | | (specify) ……………………………………………… | | | | | | | | | | | | | | | | | | | | |
| B8 | What is your caste? | | SC ⬜ | | | | BC ⬜ | | | | | | | | | | | | | | OBC ⬜ | | | | | | |
|  |  | | Other ⬜ | | | | (specify) ……………………………………………… | | | | | | | | | | | | | | | | | | | | |
| B9 | What is your current marital status? | | | | | Married ⬜  Widowed ⬜ | | | | | Separated or deserted ⬜  Divorced ⬜ | | | | | | | | | | | | | Never married ⬜ | | | |
| B10 | Do you currently live with your husband/father of your child? | | | | | | | | | | | | | | | | Yes ⬜ | | | | | | | No ⬜ | | | |
| B11 | Do you live in a Nuclear, Joint or Extended family? | | | | | | | | | | | Nuclear ⬜ | | | | | | Joint ⬜ | | | | | | Extended ⬜ | | | |
| B12 | Total household members | | | | | | | | | | | ⬜⬜ | | | | | | | | | | | |  | | | |
| B13 | ***Not to be asked to unmarried or widowed mother***  How old was your husband on his last birthday? | | | | | | | | | | | ⬜⬜ *years* | | | | | | | | | | | | Don’t know ⬜ | | | |
| B14 | ***Not to be asked to unmarried or widowed mother***  What sort of education has your husband had? | | | | | | | | | | | | | | | | | | | | | No education at all ⬜ | | | | | |
|  | Informal education ⬜ | | | | School up to class ⬜⬜ | | | | | | | | | | College ⬜⬜ | | | | | | | | | | Don’t know ⬜ | | |
| B15 | Do you own this house? | | | | | | | | | | | | Yes ⬜ | | | | | | | | | | | | | | No ⬜ |
| B16 | Type of house ***Interviewer to observe*** | | | | | | | | Pucca ⬜ | | | | Semi-pucca/semi-kacha ⬜ | | | | | | | | | | | | | | Kacha ⬜ |
| B17 | Does your household have a ration card? | | | | | | | | White ⬜ | | | | Yellow ⬜ | | | | | | Orange ⬜ | | | | | | | | No card ⬜ ***go to B19*** |
| B18 | Does the card have your name on it? | | | | | | | | Yes ⬜ | | | | No ⬜ | | | | | |  | | | | | | | | |
| B19 | Do you possess the following items?  ***Interviewer may tick more than one box*** ***Interviewer to observe and only ask for******assets that might not be seen*** | | | | | | | | | | | | | | | | | | | | | | | | | | |
|  |  | | | Mattress ⬜  Pressure cooker ⬜  Gas cylinder/chula ⬜  Stove ⬜  Chair ⬜  Cot or bed ⬜  Table ⬜  Clock ⬜  Electric fan ⬜  Grinder/mixer ⬜ | | | | | | | | | | | | | Bicycle ⬜  Radio ⬜  Sewing machine ⬜  Telephone or mobile ⬜  Refrigerator ⬜  TV ⬜  Moped, scooter or motorcycle ⬜  Car ⬜  Washing machine ⬜  Cup-board ⬜ | | | | | | | | | | |

| *Now I would like to ask you about your work, and whether you earn any money.*  *Have you done any paid work in the last 12 months?* | | | | |
| --- | --- | --- | --- | --- |
|  | 🞎 Yes | | 🞎 No | *If no, go to qx* |
| *What is your occupation, or, what kind of work do you mainly do?* | | | | |
| *Informal sector* | | *Formal sector* | | |
| 🞎 Home work: beading, cloth cutting, embroidery, tailoring, catering, pottery | | 🞎 White collar: office work, computer, telecom, call-centre | | |
| 🞎 Domestic (servant) work | | 🞎 Public sector community work: CHV, AWW, Balwadi | | |
| 🞎 Vending and stallholding: brooms, flowers, vegetables | | 🞎 Teaching: school or tutorial | | |
| 🞎 Manual labour | | 🞎 Salaried blue collar: factory, post office, hospital, catering | | |
| 🞎 Other *what? …………………………………..* | | 🞎 Health care: nursing, physio, other therapy | | |
|  | | 🞎 Other *what? …………………………………..* | | |

| *What is you husband’s occupation, or, what kind of work he mainly do?* | |
| --- | --- |
| *Informal sector* | *Formal sector* |
| 🞎 Home work: beading, cloth cutting, embroidery, tailoring, catering, pottery | 🞎 White collar: office work, computer, telecom, call-centre |
| 🞎 Domestic (servant) work | 🞎 Public sector community work: CHV, AWW, Balawadi |
| 🞎 Vending and stall holding: brooms, flowers, vegetables | 🞎 Teaching: school or tutorial |
| 🞎 Manual labour | 🞎 Salaried blue collar: factory, post office, hospital, catering |
| 🞎 Other *what? ………………………………………..* | 🞎 Health care: nursing, physio, other therapy |
|  | 🞎 Other *what? …………………………………..* |

| *Do you have access to money that you can use for your or your baby’s health?* | |
| --- | --- |
| 🞎 Yes | 🞎 No |

| *Now I would like to ask your opinion on whether women should be involved in making decisions about the home and about the health of them and their babies.*  *Do you think a woman should be involved in making decisions about:* | | | |
| --- | --- | --- | --- |
| Whether or not she works outside the home? | ⬜ Yes | ⬜ No | ⬜ Don’t know |
| What type of work she does? | ⬜ Yes | ⬜ No | ⬜ Don’t know |
| What her money should be spent on, if she has any money? | ⬜ Yes | ⬜ No | ⬜ Don’t know |
| What her husband’s money should be spent on? | ⬜ Yes | ⬜ No | ⬜ Don’t know |
| Her children’s education? | ⬜ Yes | ⬜ No | ⬜ Don’t know |
| Visits to her family or relatives? | ⬜ Yes | ⬜ No | ⬜ Don’t know |
| When she goes for ANC? | ⬜ Yes | ⬜ No | ⬜ Don’t know |
| What food to eat during pregnancy and after delivery? | ⬜ Yes | ⬜ No | ⬜ Don’t know |
| Where to deliver the baby? | ⬜ Yes | ⬜ No | ⬜ Don’t know |
| Seeking care if she is ill? | ⬜ Yes | ⬜ No | ⬜ Don’t know |
| Seeking care if the baby is ill? | ⬜ Yes | ⬜ No | ⬜ Don’t know |
| What to feed the baby? | ⬜ Yes | ⬜ No | ⬜ Don’t know |
| Whether or not to immunise the baby? | ⬜ Yes | ⬜ No | ⬜ Don’t know |
| How to take care of the baby? | ⬜ Yes | ⬜ No | ⬜ Don’t know |
| Whether she should save money? | ⬜ Yes | ⬜ No | ⬜ Don’t know |
| How and where to save money? | ⬜ Yes | ⬜ No | ⬜ Don’t know |

| *Now I would like to ask you about who makes certain decisions in your family.*  *Who usually makes decisions about:* |
| --- |

|  | Husband or other family members decide | I give my opinions but do not make the decision | My husband and I decide jointly | I alone decide |
| --- | --- | --- | --- | --- |
| Whether or not you work outside the home? | 🞎 | 🞎 | 🞎 | 🞎 |
| What type of work you do? | 🞎 | 🞎 | 🞎 | 🞎 |
| What your money should be spent on, if you have any money? | 🞎 | 🞎 | 🞎 | 🞎 |
| What your husband’s money should be spent on? | 🞎 | 🞎 | 🞎 | 🞎 |
| Your children’s education? | 🞎 | 🞎 | 🞎 | 🞎 |
| Visits to your family or relatives? | 🞎 | 🞎 | 🞎 | 🞎 |

*Now I would like to ask you questions about some other important aspects of a woman’s life. I know that some of these questions are very personal. However, your answers are important for helping to understand the condition of women in Mumbai. Let me assure you that your answers are completely confidential and will not be told to anyone and no one else will know that you were asked these questions.*

| *Sometimes a husband is annoyed or angered by things that his wife does. In your opinion, is a husband justified in hitting or beating his wife in the following situations?* | | | |
| --- | --- | --- | --- |
| If she does not fulfil his expectations of her household or childcare duties? | ⬜ Yes | ⬜ No | ⬜ Don’t know |
| If she doesn’t cook food properly? | ⬜ Yes | ⬜ No | ⬜ Don’t know |
| If she goes out without telling him? | ⬜ Yes | ⬜ No | ⬜ Don’t know |
| If she shows disrespect for in-laws? | ⬜ Yes | ⬜ No | ⬜ Don’t know |
| If he or his family are unhappy with her marriage contribution? | ⬜ Yes | ⬜ No | ⬜ Don’t know |
| If she argues with him? | ⬜ Yes | ⬜ No | ⬜ Don’t know |
| If he suspects her of being unfaithful? | ⬜ Yes | ⬜ No | ⬜ Don’t know |
| If she refuses to have sex with him? | ⬜ Yes | ⬜ No | ⬜ Don’t know |
| If her husband loves her? | ⬜ Yes | ⬜ No | ⬜ Don’t know |

| **Section C: Maternity History** | | | | | | | | | |
| --- | --- | --- | --- | --- | --- | --- | --- | --- | --- |
| C1 | **Previous pregnancies** | | | | | | | | |
| Pregnancies  *in order from first* | Mis -carriage | MTP | Birth | | Boys born alive | | Girls born alive | | |
| 1 | □ | □ | Born alive ⬜ | Born dead ⬜ | Now alive ⬜ | Now dead ⬜ | Now alive ⬜ | | Now dead ⬜ |
| 2 | □ | □ | Born alive ⬜ | Born dead ⬜ | Now alive ⬜ | Now dead ⬜ | Now alive ⬜ | | Now dead ⬜ |
| 3 | □ | □ | Born alive ⬜ | Born dead ⬜ | Now alive ⬜ | Now dead ⬜ | Now alive ⬜ | | Now dead ⬜ |
| 4 | □ | □ | Born alive ⬜ | Born dead ⬜ | Now alive ⬜ | Now dead ⬜ | Now alive ⬜ | | Now dead ⬜ |
| 5 | □ | □ | Born alive ⬜ | Born dead ⬜ | Now alive ⬜ | Now dead ⬜ | Now alive ⬜ | | Now dead ⬜ |
| 6 | □ | □ | Born alive ⬜ | Born dead ⬜ | Now alive ⬜ | Now dead ⬜ | Now alive ⬜ | | Now dead ⬜ |
| 7 | □ | □ | Born alive ⬜ | Born dead ⬜ | Now alive ⬜ | Now dead ⬜ | Now alive ⬜ | | Now dead ⬜ |
| 8 | □ | □ | Born alive ⬜ | Born dead ⬜ | Now alive ⬜ | Now dead ⬜ | Now alive ⬜ | | Now dead ⬜ |
| 9 | □ | □ | Born alive ⬜ | Born dead ⬜ | Now alive ⬜ | Now dead ⬜ | Now alive ⬜ | | Now dead ⬜ |
| 10 | □ | □ | Born alive ⬜ | Born dead ⬜ | Now alive ⬜ | Now dead ⬜ | Now alive ⬜ | | Now dead ⬜ |
| 11 | □ | □ | Born alive ⬜ | Born dead ⬜ | Now alive ⬜ | Now dead ⬜ | Now alive ⬜ | | Now dead ⬜ |
| 12 | □ | □ | Born alive ⬜ | Born dead ⬜ | Now alive ⬜ | Now dead ⬜ | Now alive ⬜ | | Now dead ⬜ |
| C2 | **Totals** |  | Born alive ⬜ | Born dead ⬜ | Now alive ⬜ | Now dead ⬜ | |  |  |
| Pregnancies | Miscarriages | MTPs | Born alive ⬜ | Born dead ⬜ | Now alive ⬜ | Now dead ⬜ | | Now alive ⬜ | Now dead ⬜ |
| ⬜ | ⬜ | ⬜ | ⬜ | ⬜ | ⬜ | ⬜ | | ⬜ | ⬜ |

| C3 | Did your previous pregnancy ended in miscarriage or MTP, how long after that you became pregnant again? | | | | | | ⬜⬜ months ⬜⬜ years | |
| --- | --- | --- | --- | --- | --- | --- | --- | --- |
|  | Yes ⬜ | ⬜⬜ months ⬜⬜ year ***Go to C6*** | | | | No ⬜ | | Only one pregnancy ⬜ ***Go to C6*** |
| C4 | How old is your last child? | | ⬜⬜ age of the previous child in completed years | | | | | |
| C5 | Where was the baby born? | | | Home ⬜ | Hospital, maternity home, health facility⬜ | | | |

| C6 | When you became pregnant this time, did you want to become pregnant at that time, would you have preferred to delay your next pregnancy, or did you not want any more children? | | | | | | | | |
| --- | --- | --- | --- | --- | --- | --- | --- | --- | --- |
|  | Wanted to become pregnant at that time ⬜ | | | | | Wanted to delay next pregnancy ⬜ | | | |
|  | Did not want any more children ⬜ | | | | | Did not think about it ⬜ | | | |
| C7 | When you became pregnant this time, did the father of the child want you to become pregnant at that time, would he have preferred to delay your next pregnancy, or did he not want any more children? | | | | | | | | |
|  | Wanted to become pregnant at that time 🞎 | | | Wanted to delay next pregnancy 🞎 | | | | | |
|  | Did not want any more children 🞎 | | | Did not think about it 🞎 | | | | | |
| C8 | When you became pregnant this time, did you want to have a son or a daughter, or did you have no preference? | | | | | | | | |
|  | Wanted a son 🞎 | | | Wanted a daughter 🞎 | | | | | No preference 🞎 |
| C9 | ***Not to be asked to unmarried or widowed mother: go to D1***  When you became pregnant this time, did the father of your child want to have a son or a daughter, or did he have no preference? | | | | | | | | Yes 🞎 |
|  | Wanted a son 🞎 | | | Wanted a daughter 🞎 | | | | | No preference 🞎 |
| C10 | ***Not to be asked to unmarried or widowed mother: go to D1***  Have you and your husband ever used any family planning method? | | | | | | | | |
|  | Yes 🞎 | | No 🞎 ***Go to D1*** | | | | |  | |
| C11 | Which method have you used in the last 3 years? ***Do not read out options Interviewer may tick more than one*** | | | | | | | | |
|  | Breastfeeding 🞎  Oral contraceptive pill 🞎  Norplant 🞎  Depo provera 🞎 | Intrauterine device (coil, copper T, loop) 🞎  Condoms 🞎  Safe period 🞎  Withdrawal 🞎 | | | | | Traditional medicine 🞎  Tubectomy 🞎  Vasectomy 🞎  Emergency contraception 🞎  Others 🞎……………… | | |
|  | (Go to C13 if method used is condom, withdrawal or vasectomy) | | | | | | | | |
| C12 | Does your husband know the method you use? | | | | Yes 🞎 | | No 🞎 | | |
| C13 | Are you satisfied with the method? | | | | Yes 🞎 | | No 🞎 | | |

| *If you use a method of family planning in the future, will you tell your husband?* | | |
| --- | --- | --- |
|  | 🞎 Yes | 🞎 No |

***Thank you for answering those questions.***

***Now I am going to ask you about antenatal care in the pregnancy you have just had.***

|  | **Section D: Antenatal care** | | | | | | | | | | | | | | | | | | |
| --- | --- | --- | --- | --- | --- | --- | --- | --- | --- | --- | --- | --- | --- | --- | --- | --- | --- | --- | --- |
| D1 | Did any health worker register your pregnancy? | | | | | | | | | | | | | | | | | | No 🞎 |
|  |  | Yes… | | BMC CHV 🞎 | | | | | | | ICDS AWW 🞎 | | | | | | | | NGO CHV 🞎 |
|  |  | | | Specify name of NGO/CBO | | | | | | | ……………………………… | | | | | | | | |
| D2 | Is there an antenatal clinic at your nearest BMC health post? | | | | | | | | | | Yes 🞎 | | | | No 🞎 | | | | Don’t know 🞎 |
| D3 | Did you go for an antenatal check-up during this pregnancy? | | | | | | | | | | Yes 🞎 | | | | ***Go to D5*** | | | | No 🞎 |
| D4 | Why not? ***Interviewer may tick more than one box. Do not read out the options. Tick options depending on what respondent says.*** | | | | | | | | | | | | | | | | | | |
| No problem 🞎  Did not see need 🞎  No time to go 🞎 | | | | | | Family did not allow 🞎  Nobody to manage children or home 🞎  Nobody to accompany 🞎 | | | | | | | | Too far away 🞎  Cost of service 🞎  Not customary 🞎  Afraid of hospital 🞎 | | | | | |
|  | ***If no antenatal care go to D12*** | | | | | | | | | | | | | | | | | | |
| D5 | What was the reason for your first antenatal care visit? | | | | | | | Check-up 🞎 | | | | Problem 🞎 | | | | | (what) …………… | | |
| D6 | Where did you go for antenatal care? | | | | | | | | | | | | | | | |  | | |
|  | Health Post 🞎 | | | | | | Maternity home 🞎 | | General hospital 🞎 | | | | | | | | Large hospital 🞎 | | |
|  | Private facility 🞎 | | Other 🞎 | | | | Government hospital 🞎 | | Outside Mumbai 🞎 | | | | | | | | UHC 🞎 | | |
|  | Name and location of institution ……………………………………………………………….. | | | | | | | | | | | | | | | | | | |
| D7 | In which month of pregnancy did you go for your first antenatal check-up?  ***Write 1 if less than 1 month*** | | | | | | | | | | | | 🞎 *months* ***if < 7 go to D9*** | | | | | | |
| D8 | What were the reasons for waiting until this stage of pregnancy? ***Interviewer may tick more than one box Do not read out the options. Tick options depending on what respondent says.*** | | | | | | | | | | | | | | | | | | |
| No problem 🞎  Did not see need 🞎  No time to go 🞎 | | | | | Family did not allow 🞎  Nobody to manage children or home 🞎  Nobody to accompany 🞎 | | | | | | | | | | | Too far away 🞎  Cost of service 🞎  Not customary 🞎  Afraid of hospital 🞎 | | | |
| D9 | How many antenatal check-ups did you have? | | | | | | | | | | | | | | | | | | |
|  | In first 3 months 🞎 times | | | | In middle 3 months 🞎 times | | | | | In last 3 months 🞎 times | | | | | | | | total 🞎 times | |

| D10 | During antenatal care, did this check happen………………… ***Read out options one by one*** | | | | | | | | | | | | | | | | | | |
| --- | --- | --- | --- | --- | --- | --- | --- | --- | --- | --- | --- | --- | --- | --- | --- | --- | --- | --- | --- |
| Measured your weight 🞎  Measured your height 🞎  Examined your abdomen 🞎  Measured your blood pressure (with a cuff) 🞎  Took a urine sample 🞎  Took a blood sample 🞎  Did an internal examination 🞎 | | | | | | | | | Did an ultrasound scan 🞎  Gave you an injection in the arm to prevent tetanus (TT) 🞎  Gave you iron supplements 🞎  Discussion on having a HIV test 🞎  Did an HIV test 🞎  Collected the result of the test 🞎  Doctor advised on the result of the test 🞎  Gave you a mother and child card 🞎 | | | | | | | | | | |
| D11 | What was the due date for delivery (**EDD)?** | | | | | | | | _ _ / _ _ / _ _ _ _ *day/month/year* | | | | | | | | | Don’t know 🞎 | |
| D12 | Did you take iron tablets from the fifth month of the pregnancy? | | | | | | | | | | | | | | | | | | No 🞎 |
|  | Yes: 1 packet 🞎 | | | | | | | Yes: 2 packets 🞎 | | | | | | Yes: 3 packets or more 🞎 ***go to D14*** | | | | | |
| D13 | What were your reasons for not taking the full course of iron tablets? | | | | | | | | | | | | | | | | | | |
|  | Had side-effects 🞎 | | | | Did not see need 🞎 | | | | | Lost or misplaced tablets 🞎 | | | | | | | Someone else took tablets 🞎 | | |
|  | Other 🞎 | | | | (why) ……………………… | | | | | | | | | | | | | | |
| D14 | While you were pregnant, did a health worker visit you at home to discuss issues about the pregnancy? | | | | | | | | | | | | | | | | | | |
|  |  |  | | | | | | | | | | | Yes 🞎 | | | No 🞎 ***go to D16*** | | | |
| D15 | Who visited you? | | | | | | | | | | | | | | | | | | |
|  | BMC CHV 🞎 | | | ICDS AWW 🞎 | | | NGO CHV 🞎 | | | | | Specify name of NGO/CBO 🞎 ……………….. | | | | | | | |
| D16 | Did the amount of rest you took during pregnancy change from before you were pregnant? | | | | | | | | | | | | | | | | | | |
| In first 3 months | | | More 🞎  Same 🞎  Less 🞎 | | | In middle 3 months | | | | | | More 🞎  Same 🞎  Less 🞎 | | | In last 3 months | | | | More 🞎  Same 🞎  Less 🞎 |
| D17 | Did the amount of physical work you did during pregnancy change from before you were pregnant? | | | | | | | | | | | | | | | | | | |
| In first 3 months | | | More 🞎  Same 🞎  Less 🞎 | | | In middle 3 months | | | | | More 🞎  Same 🞎  Less 🞎 | | | | In last 3 months | | | | More 🞎  Same 🞎  Less 🞎 |
| D18 | Did the amount of food you ate during pregnancy change from before you were pregnant? | | | | | | | | | | | | | | | | | | |
| In first 3 months | | | More 🞎  Same 🞎  Less 🞎 | | | In middle 3 months | | | | | More 🞎  Same 🞎  Less 🞎 | | | | In last 3 months | | | | More 🞎  Same 🞎  Less 🞎 |

| *When a woman is pregnant or has just had a baby, some behaviours can be healthy or harmful. I would like to ask your opinion about some behaviours.*  *During pregnancy, or after having a baby, do you think a woman should:* | | | |
| --- | --- | --- | --- |
| Go for ANC in the first 3 months of pregnancy? | 🞎 Yes | 🞎 No | 🞎 Don’t know |
| Take more rest during pregnancy than before pregnancy? | 🞎 Yes | 🞎 No | 🞎 Don’t know |
| Work more during pregnancy than before pregnancy? | 🞎 Yes | 🞎 No | 🞎 Don’t know |
| Eat more food during pregnancy than before pregnancy? | 🞎 Yes | 🞎 No | 🞎 Don’t know |
| Have a home birth if she has problems during pregnancy? | 🞎 Yes | 🞎 No | 🞎 Don’t know |
| Give her baby the first milk (colostrum)? | 🞎 Yes | 🞎 No | 🞎 Don’t know |
| Feed her baby things other than breastmilk? | 🞎 Yes | 🞎 No | 🞎 Don’t know |

| *When you were pregnant, and since you had the baby, who made decisions about:* |
| --- |

|  | Husband or other family members decided | I gave my opinions but did not make the decision | My husband and I decided jointly | I alone decided | Did you agree with the decision? | Did you negotiate about the decision? |
| --- | --- | --- | --- | --- | --- | --- |
| When you went for ANC? | 🞎 | 🞎 | 🞎 | 🞎 | 🞎 Yes 🞎 No | 🞎 Yes 🞎 No |
| What food you ate during your pregnancy and afterwards? | 🞎 | 🞎 | 🞎 | 🞎 | 🞎 Yes 🞎 No | 🞎 Yes 🞎 No |
| Where you delivered the baby? | 🞎 | 🞎 | 🞎 | 🞎 | 🞎 Yes 🞎 No | 🞎 Yes 🞎 No |
| Seeking care if you were ill? | 🞎 | 🞎 | 🞎 | 🞎 | 🞎 Yes 🞎 No | 🞎 Yes 🞎 No |
| Seeking care if your baby was ill? | 🞎 | 🞎 | 🞎 | 🞎 | 🞎 Yes 🞎 No | 🞎 Yes 🞎 No |
| Whether or not you immunised your baby? | 🞎 | 🞎 | 🞎 | 🞎 | 🞎 Yes 🞎 No | 🞎 Yes 🞎 No |
| How you take care of the baby? | 🞎 | 🞎 | 🞎 | 🞎 | 🞎 Yes 🞎 No | 🞎 Yes 🞎 No |

*Sometimes a woman might make decisions about her or her baby’s health, but cannot carry them out because her husband or family do not allow it.*

*During your pregnancy or after your delivery, did you want to do any of the following, but were prevented from doing so by your husband or other family member?*

| Wanted to go for ANC? | 🞎 Yes | 🞎 No |
| --- | --- | --- |
| Wanted to take more rest? | 🞎 Yes | 🞎 No |
| Wanted to reduce your household chores? | 🞎 Yes | 🞎 No |
| Wanted to visit family or friends? | 🞎 Yes | 🞎 No |
| Wanted to go to community meetings? | 🞎 Yes | 🞎 No |
| Wanted to go to a health facility if you were ill? | 🞎 Yes | 🞎 No |
| Wanted to deliver your baby at a health facility? | 🞎 Yes | 🞎 No |
| Wanted to take your baby to a health facility? | 🞎 Yes | 🞎 No |
| Wanted to give your baby your first milk (colostrum?) | 🞎 Yes | 🞎 No |
| Wanted to immunise your baby? | 🞎 Yes | 🞎 No |

|  | **Section E: Illness during pregnancy** | | | | | | | | | | | | | | | | | | | | | | | |
| --- | --- | --- | --- | --- | --- | --- | --- | --- | --- | --- | --- | --- | --- | --- | --- | --- | --- | --- | --- | --- | --- | --- | --- | --- |
| E1 | Women have certain problems during pregnancy. For example, the baby may stop moving or your legs may be swollen. What sort of problems did you have while you were pregnant? ***Interviewer may tick more than one box*** ***Do not read out the options. Tick options depending on what respondent says.*** | | | | | | | | | | | | | | | | | | | | | | | |
|  | No problem at all  | | | | | | | | | | | | ***go to F1*** | | | | | | | | | | | |
|  | Baby stopped moving 🞎  Swollen legs 🞎  Convulsion/fit/seizure/loss of consciousness 🞎  Vaginal bleeding 🞎  Waters leaked 🞎  Jaundice 🞎  Difficulty seeing at night 🞎  Breathless doing normal household tasks 🞎  Burning when passing urine 🞎  Frequent need to pass urine 🞎  Abnormal vaginal discharge 🞎  Fever 🞎  Vomit/Diarrhoea 🞎 | | | | | | | | | | | | Cough 🞎  Rash 🞎  Backache 🞎  Exhausted/very tired/weak 🞎  Headaches or dizziness 🞎  Nausea, bloating or indigestion 🞎  Constipation 🞎  Abdominal pain 🞎  Cramps 🞎  Waist pain 🞎  High or low BP 🞎  Anaemia 🞎  Prolapse of uterus 🞎 | | | | | | | | | | | |
|  | Other 🞎 | | | | | | | | | | | | (specify) ………………………………… | | | | | | | | | | | |
|  | ***If only one problem go to E3*** | | | | | | | | | | | |  | | | | | | | | | | | |
| E2 | Which one was the most dangerous and in a paisa scale how you are going to rate it? *(25 paise, 50 paise, 75 paise)* | | | | | | | | | | | | ………………*paise* | | | | | | | | | | | |
| E3 | Did you try any home remedies to treat the illness at home? | | | | | | | | | | | | Yes 🞎 | | | No 🞎 | | | | | | ***go to E6*** | | |
| E4 | What did you do? | | | ……………………………………………………………………………………………………………..………..………..………..………..………..………………… | | | | | | | | | | | | | | | | | | | | |
| E5 | How long was it from the time you recognized you had a problem until you first treated it at home? | | | | | | | | | | | | | | | | | | | | | | | |
|  | ⬜⬜ *mins* or ⬜⬜ *hours* or ⬜⬜ *days* | | | | | | | | | | | | | | | | | | | | | | | |
| E6 | Did you seek care from someone outside the home? | | | | | | | | | | | Yes 🞎 | | | | | |  | | | No 🞎 | | | |
|  | ***If she did not seek care from someone outside the home go to F section*** | | | | | | | | | | | | | | | | | | | | | | | |
| E7 | Where did you seek help? | | | | | |  | | | | | | | | | | | | | | | | | |
|  | Health Post 🞎 | Maternity home 🞎 | | | | | | | | | | | | | General hospital 🞎 | | | | | Very large hospital 🞎 | | | | |
|  | Private facility 🞎 | Other 🞎 | | | | Government hospital 🞎 | | | | | | | | | Outside Mumbai 🞎 | | | | | UHC 🞎 | | | | |
|  | Name and location of institution …………………………………………………………………….……….…. | | | | | | | | | | | | | | | | | | | | | | | |
| E8 | How long was it from the time you recognised you had a problem until you got treatment? | | | | | | | | | | | | | | | | | | | | | | | |
|  |  | | | | | | | ⬜⬜ *mins* or ⬜⬜ *hours* or ⬜⬜ *days* | | | | | | | | | | | | | | | | |
| E9 | Were you referred or transferred for further treatment? | | | | | | | | | | | | | | | | | | | | | | | |
|  | Sent from gate or OPD 🞎 | | | | | | | | | Transferred after admission 🞎 | | | | | | | | | | | | No  ***go to E12*** | | |
| E10 | Did you go to the place to which they referred or transferred you? | | | | | | | | | | | | | | | | Yes 🞎 | | | | | No  ***go to E12*** | | |
| E11 | Where did you go? | | | | | | | | | | | | | |  | | | | |  | | | | |
|  | Health Post 🞎 | | | | Maternity home 🞎 | | | | | | | | | | General hospital 🞎 | | | | | Very large hospital 🞎 | | | | |
|  | Private facility 🞎 | Other 🞎 | | | | Government hopsital 🞎 | | | | | | | | | Outside Mumbai 🞎 | | | | | UHC 🞎 | | | | |
|  | Name and location of institution ……………………………………………………….……….……….………… | | | | | | | | | | | | | | | | | | | | | | | |
| E12 | Did you seek care from someone else? | | | | | | | | | | | | | Yes 🞎 | | | | No 🞎 | | | | | | |
|  | ***If she did not seek care from someone else go to E15*** | | | | | | | | | | | | | | | | | | | | | | | |
| E13 | Where did you seek help? | | | | | |  | | | | | | | | | | | | | | | | | |
|  | Health Post 🞎 | | Maternity home 🞎 | | | | | | General hospital 🞎 | | | | | | | | | | Very large hospital 🞎 | | | | | |
|  | Private facility 🞎 | | Other 🞎 | | | | | | Government hospital 🞎 | | | | | | | | | | Outside Mumbai 🞎 | | | | | UHC 🞎 |
|  | Name and location of institution ……………………………………………………….……….……….………… | | | | | | | | | | | | | | | | | | | | | | | |
| E144 | How long was it from the time you recognised you had a problem until you got treatment? | | | | | | | | | | | | | | | | | | | | | | | |
|  |  | | | | | | | | | | | _ _ *mins* or _ _ *hours* or _ _ *days* | | | | | | | | | | | | |
| E15 | If you had the problem again, where would you go? | | | | | | | | | | Same place 🞎 | | | | | | Other place 🞎 | | | | | | Nowhere 🞎 | |

|  | **Section F: Current delivery** | | | | | | | | | | | | | | | | | |
| --- | --- | --- | --- | --- | --- | --- | --- | --- | --- | --- | --- | --- | --- | --- | --- | --- | --- | --- |
| F1 | Was your delivery in Mumbai or outside Mumbai? | | | | | In Mumbai 🞎 | | | | | | | | | | Outside Mumbai 🞎 | | |
| F2 | In which place was your delivery? | | | | | | | | | | | | | | | | | |
|  | Home 🞎 | | | | | | On way to facility 🞎 | | | | | | | | | | | |
|  | Government Hospital 🞎 | Maternity home 🞎 | | | | | | | General hospital 🞎 | | | | | | | | | Very large hospital 🞎 |
|  | Private facility 🞎 | Government hospital 🞎 | | | | | | | UHC 🞎 | | | | | Other 🞎 | | | | (specify) …………… |
|  | Name and location of institution ………………………………………………………….……….………. | | | | | | | | | | | | | | | | | |
| F3 | On what date was your delivery? | | | | | _ _ / _ _ / _ _ _ _ *day/month/year* | | | | | | | | | | | | |
| F4 | What time was your delivery? | | | | | ⬜⬜⬜⬜ ***time according to 24 hr clock*** | | | | | | | | | | | | |
| F5 | Had you registered your name in advance for delivery at a health facility? | | | | | | | | | Yes 🞎 | | | | | | | | No 🞎 ***go to F10*** |
| F6 | Where did you register your name for delivery? | | | | | | | | |  | | | | | | | |  |
|  | Maternity home 🞎 | | General hospital 🞎 | | | | | | | Very large hospital 🞎 | | | | | | | | Private facility 🞎 |
|  | Other 🞎 | | Government hospital 🞎 | | | | | | | Outside Mumbai 🞎 | | | | | | | | UHC 🞎 |
|  | Name and location of institution ………………………………………………………………….. | | | | | | | | |  | | | | | | | |  |
| F7 | In which month of pregnancy did you register your name for delivery? ***Write 1 if less than 1 month*** | | | | | | | | | | | | | | | | | __ *months* |
|  | **If home births, go to F14** | | | | | | | | | |  | | | | | | |  |
| F8 | Did you deliver in the same facility where you registered your name for delivery? | | | | | | | | | | Yes 🞎 ***Go to F10*** | | | | | | | No 🞎 |
| F9 | Why did you not deliver in the facility where you registered? | | | | | | | | | | ***Interviewer may tick more than one box*** | | | | | | | |
| I | **Decided to go somewhere else ** | | | |  | | | | | |  | | | | | | | |
|  | 2 child norm 🞎 | | | | Too far 🞎 | | | | | | | | Renovation/strike/closed/doctor unavailable 🞎 | | | | | |
|  | Poor opinion of registered facility (care, attitude, hygiene, services, afraid) 🞎 | | | | Good opinion of other facility 🞎 | | | | | | | | Family/friend/doctor advised 🞎 | | | | | |
|  | No one to accompany 🞎 | | | | Did not go there because it would cost too much 🞎 | | | | | | | | Went there but found out it would cost too much 🞎 | | | | | |
| II | **Not enough time to reach** 🞎 | | | |  | | | | | | | | | | | | | |
| III | **Went to mother’s home or village** 🞎 | | | |  | | | | | | | | | | | | | |
| IV | **Moved/shifted house** 🞎 | | | |  | | | | | | | | | | | | | |
| V | **Sent away before admission** 🞎 | | | | Medical problem/needed CS 🞎 | | | | | | | | | | Told to return at a later stage 🞎 | | | |
|  | Renovation/strike/closed/doctor unavailable 🞎 | | | | | | | | | | | | | | Refused admission 🞎 | | | |
| VI | **Transferred after admission** 🞎 | | | | Medical problem/needed CS 🞎 | | | | | | | | | | Lack of equipments/facilities (oxygen, blood, electricity, baby care) 🞎 | | | |
| VII | **Others (specify**)………………………. | | | | | | | | |  | | | |  | | | | |
| F10 | Do you have the hospital records? | | | | | | | Yes 🞎 | | | | | | | | | No 🞎 | |
| F11 | Who accompanied you at the time of delivery? | | | | | | | | | | | | | | | |  | |
|  | Husband 🞎 | | | | Friend/neighbour 🞎 | | | | | | | | | | | | Sister 🞎 | |
|  | Mother-in-law 🞎 | | | | Anganwadi Helper 🞎 | | | | | | | | | | | | Sister-in-law 🞎 | |
|  | Mother 🞎 | | | | Other 🞎 (specify) …………………………………….… | | | | | | | | | | | | | |
| F 12 | Have you paid any fine for having more than 2 children? | | | | | | | | | | | Yes 🞎 | | | | | No 🞎 | |
|  | Other 🞎 (specify) …………………………………………………… | | | | | | | | | | | | | | | | | |
| F13 | Have you heard of any scheme to give you some money if you have your baby at a health facility? | | | | | | | | | | | | | | | | | |
|  | Yes 🞎 | | | No 🞎 ***Go to G or H section depending on home or hospital birth*** | | | | | | | | | | | | | | |
| F14 | Were you offered any money under such a scheme? | | | | | | | | | | | | | | | | | |
|  | Yes 🞎 | | | | | | | No 🞎 ***Go to G1*** | | | | | | | | | Home birth 🞎 ***Go to G1*** | |
| F15 | Have you received the money? | | | | | | | Yes 🞎 | | | | | | | | | No 🞎 | |
| F16 | If yes, how much did you get? | | | | | | | Rs. _____ | | | | | | | | |  | |

**Choose section G or H depending on home or hospital birth**

|  | **Section G: Home/on the way delivery** | | | | | | | | |
| --- | --- | --- | --- | --- | --- | --- | --- | --- | --- |
| G1 | Why did you give birth at home? …………………………………………………………………………….. | | | | | | | | |
| G2 | Who was the main person who assisted you in delivering your baby? | | | | | | | | |
|  | Friend or relative 🞎 | Dai 🞎 | | CHV 🞎 | | Nobody assisted 🞎  ***Go to G5*** | | | |
|  | Other 🞎 | (who) …………………………………… | | | | | | | |
| G3 | Did the person who helped wash her hands with soap before the delivery? | | | | | | Yes 🞎 | No 🞎 | Don’t know 🞎 |
| G4 | Did the person who helped wear gloves during the delivery? | | | | | | | | |
|  | Yes: new gloves 🞎 | | Yes: old gloves 🞎 | | No 🞎 | | | Don’t know 🞎 | |

| G5 | What was the cord cut with? | | | | | | Blade 🞎 | | | | | | Knife 🞎 | | | | | Scissors 🞎 | | | | | Don’t know 🞎 | | |
| --- | --- | --- | --- | --- | --- | --- | --- | --- | --- | --- | --- | --- | --- | --- | --- | --- | --- | --- | --- | --- | --- | --- | --- | --- | --- |
|  |  | |  | | | | Other 🞎 (what) ……………..……………………… | | | | | | | | | | | | | | | | | | |
| G6 | Was the cutting implement brand new, boiled or used? | | | | | | | | | | | | | | | | | | | | | | | | |
|  | Absolutely new 🞎 | | | | | Boiled 🞎 | | | | | | | Used 🞎 | | | | | | | | | Don’t know 🞎 | | | |
| G7 | What was the umbilical cord tied with? | | | | | | | | | | | |  | | | | | | | | |  | | | |
|  | Clamp 🞎 | | | | | Boiled thread 🞎 | | | | | | | Unboiled thread 🞎 | | | | | | | | | Don’t know 🞎 | | | |
|  | Other 🞎 | | | | | | | | | | | | (specify) ………………..……….………. | | | | | | | | | | | | |
| G8 | What was applied on the cord stump after it was cut? | | | | | | | | | | | | | | | | | | | | | | | | |
|  | Nothing 🞎 | | | Kerosene 🞎 | | | | | | | Oil 🞎 | | | | Turmeric 🞎 | | | | | | Antiseptic cream 🞎 | | | | |
|  | Boric powder 🞎 | | | | | | | | | | Other 🞎 (specify) ……….……….………. | | | | | | | | | | | | | | |
| G9 | Women have certain problems during delivery. For example, the baby might get stuck or the placenta might not come out.  What sort of problems did you have while giving birth? *Interviewer may tick more than one box* ***Do not read out the options. Tick options depending on what respondent says.*** | | | | | | | | | | | | | | | | | | | | | | | | |
|  | No problem at all  | | | | | | | | | | | | | ***go to I1*** | | | | | | | | | | | |
|  | Labour longer than 24 hours 🞎  Baby was stuck 🞎  Convulsion/fit/seizure/loss of consciousness 🞎  So much vaginal bleeding you thought you would die 🞎 | | | | | | | | | | | | | Placenta stuck longer than half hour 🞎  Tear in birth canal 🞎  Fever 🞎  Exhaustion 🞎 | | | | | | | | | | | |
|  | Other 🞎 | | | | | | | | | | | | | (what) ………………………….. | | | | | | | | | | | |
|  | ***If only one problem go to G11*** | | | | | | | | | | | | |  | | | | | | | | |  | | |
| G10 | Which one was the most dangerous and in a paisa scale how you are going to rate it? *(25 paise, 50 paise, 75 paise)* | | | | | | | | | | | | | ………………*paise* | | | | | | | | |  | | |
|  |  | | | | | | | | | | | | |  | | | | | | | | |  | | |
| G11 | Did you try any home remedies to treat the illness at home? | | | | | | | | | | | | | Yes 🞎 | | | | | No 🞎 ***go to G14*** | | | | | | |
| G12 | What sort of treatment did you do? | | | | | | | ……………………………………………………………………………… | | | | | | | | | | | | | | | | | |
| G13 | How long was it from the time you recognized you had a problem until you first treated it at home? | | | | | | | | | | | | | | | | | | | | | | | | |
|  |  | | | | | | | | | | | _ _ *mins* or _ _ *hours* or _ _ *days* | | | | | | | | | | | | | |
| G14 | Did you seek care from someone outside the home? | | | | | | | | | | | Yes 🞎 | | | | | | | | No 🞎 | | | | | |
|  | ***If she did not seek care from someone outside the home go to I section*** | | | | | | | | | | | | | | | | | | | | | | | | |
| G15 | Where did you seek help? | | | |  | | | | | | | | | | | | | | | | | | | | |
|  | Health Post 🞎 | Maternity home 🞎 | | | | | General hospital 🞎 | | | | | | | | | | Very large hospital 🞎 | | | | | | | | |
|  | Private facility 🞎 | Other 🞎 | | | | | Government hospital 🞎 | | | | | | | | | | Outside Mumbai 🞎 | | | | | | | | UHC 🞎 |
|  | Name and location of institution …………………………………………………………… | | | | | | | | | | | | | | | | | | | | | | | | |
| G16 | How long was it from the time you recognised you had a problem until you got treatment? | | | | | | | | | | | | | | | | | | | | | | | | |
|  |  | | | | | | | | | _ _ *mins* or _ _ *hours* or _ _ *days* | | | | | | | | | | | | | | | |
| G17 | If you had the problem again, where would you go? | | | | | | | | Same place 🞎 | | | | | | | Other place 🞎 | | | | | | | | Nowhere 🞎 | |

| **Section H: Facility births** | | | | | |
| --- | --- | --- | --- | --- | --- |
| H1 | How was your baby delivered?  ***Check baby birth card or discharge summary*** | | Normal 🞎  Forceps 🞎 | | Vacuum 🞎  Operation, caesarean 🞎 |
| H2 | **Women have certain problems during delivery. For example, the baby might get stuck or the placenta might not come out.**  What sort of problems did you have during the delivery? *Do not read out the options.* ***Interviewer may tick more than one box. Allow the woman to answer and tick options accordingly.*** | | | | |
|  | No problem at all 🞎 |  | | | |
|  | Labour longer than 24 hours 🞎  Baby was stuck 🞎  Required vacuum 🞎  Required forceps 🞎  Required caesarean section 🞎  Convulsion/fit/seizure/loss of consciousness 🞎 | So much vaginal bleeding she thought she would die 🞎  Placenta stuck longer than half hour 🞎  Tear in birth canal 🞎  Fever 🞎  Exhaustion 🞎 | | | |
|  | Other 🞎 | (what) ……………………………………………….. | | | |
| H3 | In what ways did you receive support during delivery from family or friends? | | |  | |
|  | Provided no support 🞎  Provided money 🞎  Remained at facility for duration 🞎  Visited regularly 🞎 | Sent clothes 🞎  Sent food 🞎  Obtained medicines 🞎  High blood pressure 🞎 | | | |
|  | Other 🞎 | (what) ……………………………………………….. | | | |

|  | **Section I: Newborn** | | | | | | | | | | | | | | | | |
| --- | --- | --- | --- | --- | --- | --- | --- | --- | --- | --- | --- | --- | --- | --- | --- | --- | --- |
| I1 | Was the baby a multiple pregnancy? | | | Yes  ***Fill out a separate section for each baby*** | | | | | | | | | | | | | No 🞎 |
| I2 | Is your baby a boy or a girl? | | | | Boy 🞎 | | | | | | | | | | | | Girl 🞎 |
| I3 | After how many completed months of pregnancy was your baby born?  ***Use available papers to help answer the question*** | | | | | | | | | | | | | | __ *months* | | |
| I4 | How big was your baby at birth? | Very small/smaller than usual 🞎  Average/normal size 🞎 | | | | | | | | | | | Very large/bigger than usual 🞎  Don’t know 🞎 | | | | |
| I5 | Was your baby weighed at birth? | Yes 🞎 | | | | | | | | | | | No 🞎 ***go to I7*** | | | | |
| I6 | Birth weight  ***Use available papers*** | _ _ _ _ *grams* | | | | | | | | | | | Don’t know 🞎 | | | | |
| I7 | How long after birth was your baby given to you? | | | | | | | | _ _ *mins* or _ _ *hours* or _ _ *days* | | | | | | | | |
| I8 | How long after birth was your baby bathed/ sponged? | | | | | | | | Bathed 🞎 | | Sponged 🞎 | | | | | Don’t know 🞎 | |
| I8 (a) | Bathed | | | | | | | | _ _ *mins* or _ _ *hours* or _ _ *days* | | | | | | | | |
| I8 (b) | Sponged | | | | | | | | _ _ *mins* or _ _ *hours* or _ _ *days* | | | | | | | | |
| I9 | Was your baby examined by a healthcare provider after birth?  ***Use available papers*** | | | | | | | | | Yes 🞎 | | | | | | No 🞎 ***go to I12*** | |
| I10 | How long after birth was your baby examined? | | | | | | _ _ *mins* or _ _ *hours* or _ _ *days* | | | | | | | | | | |
| I11 | Who examined your baby? | | | | | | Doctor 🞎 | | | Nurse 🞎 | | | | | | Dai 🞎 | |
|  |  | | | | | | Other 🞎 | | | (who) ……………………………… | | | | | | | |
| I12 | What was the very first thing fed to your baby? | | Breast milk 🞎  Bottle milk 🞎 | | | | | Honey 🞎  Sugar water 🞎  Glucose water 🞎 | | | | | | Cow, buffalo, goat milk 🞎  Caster oil 🞎  Other woman’s milk 🞎 | | | |
|  |  | | Other 🞎 | | | | | (specify) ………………………………… | | | | | | | | | |
| I13 | Have you breastfed your baby? | | | | | | | Yes 🞎 | | | | No 🞎 ***go to I17*** | | | | | |
| I14 | When did you first breastfeed your baby? | | | | | | _ _ *mins* or _ _ *hours* or _ _ *days* | | | | | | | | | | |
| I15 | Did you feed your baby the first milk that you produced, or did you remove it and throw it away? | | | | | | | | | | | | | | | | |
|  |  | | | | | Feed milk 🞎 | | | | | | Discarded milk 🞎 | | | | | |
| I16 | Are you still breastfeeding your baby | | | | | Yes 🞎 ***go to I18*** | | | | | | No 🞎 | | | | | |

| 17 | Why are you not breastfeeding your baby? *Do not read out the options.* ***Interviewer may tick more than one box. Allow the woman to answer and tick options accordingly.*** | | | | | | | | | | | | | | | | |
| --- | --- | --- | --- | --- | --- | --- | --- | --- | --- | --- | --- | --- | --- | --- | --- | --- | --- |
|  |  | | | Breast problems 🞎  Household duties 🞎  Workplace norm 🞎  Baby’s stomach not full 🞎 | | | | | | Cracked nipple 🞎  Breast tenderness 🞎  Mother unwell 🞎  Baby unwell 🞎  No breast milk 🞎 | | | | | | | |
|  |  | | | | | | Other 🞎 | | | (what) …………………………. | | | | | | | |
| I18 | Apart from just after birth, have you given your baby something other than breast milk? ***If not breastfeeding she will have done so*** | | | | | | | | | | | | | Yes 🞎 | No 🞎 ***go to I22*** | | |
| I19 | When did you first give something other than breast milk? | | | | | | | | | | | _ _ *mins* or _ _ *hours* or _ _ *days* | | | | | |
| I20 | What was it?  ***Interviewer may tick more than one box*** | | | | | | | Formula milk 🞎  Cow, buffalo, goat milk 🞎 | | | | | | Other woman’s milk 🞎 | | | |
|  |  | | | | | | | Other | | | | | | (what) ……………….. | | | |
| I21 | Why did you give something other than breast milk? *Do not read out the options.* ***Interviewer may tick more than one box. Allow the woman to answer and tick options accordingly****.* | | | | | | | | | | | | | | | | |
|  |  | | Breast problems 🞎  Household duties 🞎  Workplace norm 🞎  Baby’s stomach not full 🞎 | | | | | | | | | | | Cracked nipple 🞎  Breast tenderness 🞎  Mother unwell 🞎  Baby unwell 🞎  No breast milk 🞎 | | | |
|  |  | | | | | Other 🞎 | | | | | | | | (what) …………………… | | | |
| I22 | Have you given your baby the following?  ***Interviewer may tick more than one box*** | | | | | | | | Balghuti 🞎  Gripe water 🞎  Jeera water 🞎 | | | | | Vitamins 🞎  Sauf water 🞎 | | | |
| Other  | | | | | | | | | (specify) ………………………………….. | | | | | | | | |
| I23 | Has your baby been given the BCG immunisation? | | | | | | | | | | Yes 🞎 | | No 🞎 | | | | Don’t know 🞎 |
| I24 | Has your baby been given the first polio immunisation? | | | | | | | | | | Yes 🞎 | | No 🞎 | | | | Don’t know 🞎 |
| I25 | Do you have the baby’s birth certificate? | | | | | | | | | | Yes  ***go to J1*** | | | | | | No 🞎 |
| I26 | If no, why? | Home birth 🞎 | | | Did not feel the need 🞎 | | | | | | Did not know the procedure 🞎 | | | | | Will get it later 🞎 | |
|  | Given it for registration 🞎 | | | | Has been told to come afterwards 🞎 | | | | | | Others (specify) ……………………….. | | | | | | |

|  | **Section J: Newborn illness** | | | | | | | | | | | | | | | | | | | | | | | | | | | | |
| --- | --- | --- | --- | --- | --- | --- | --- | --- | --- | --- | --- | --- | --- | --- | --- | --- | --- | --- | --- | --- | --- | --- | --- | --- | --- | --- | --- | --- | --- |
| J1 | Babies have certain problems after birth. For example, the baby may not cry at birth or have difficulty breathing. What sort of problems has your baby had? ***Do not read out the options****.* ***Interviewer may tick more than one box. Allow the woman to answer and tick options accordingly.*** | | | | | | | | | | | | | | | | | | | | | | | | | | | | |
|  | No problem at all 🞎 | | | | | | | | | | | | | | | | | ***go to K1*** | | | | | | | | | | | |
|  | Did not cry at birth 🞎  Difficulty breathing 🞎  Difficulty breastfeeding 🞎  Small/low birth weight 🞎  Weak and lethargic 🞎  Repeated vomiting 🞎  Convulsion/fit/seizure 🞎  Unconscious 🞎  Diarrhoea 🞎  Redness around umbilicus 🞎  Congenital abnormality 🞎 | | | | | | | | | | | | | | | | | Sticky eye 🞎  White deposit in mouth 🞎  Excessive crying 🞎  Abdominal distension 🞎  Lack of movement 🞎  Skin rash 🞎  Jaundice 🞎  Cough 🞎  Fever 🞎  Cold 🞎  Constipation 🞎 | | | | | | | | | | | |
|  | Other  | | | | | | | | | | (what) ………………………………………………… | | | | | | | | | | | | | | | | | | |
|  | ***If only one problem go to G11*** | | | | | | | | | |  | | | | | | | |  | | | | | | |  | | | |
| J2 | Which one was the most dangerous and in a paisa scale how you are going to rate it? *(25 paise, 50 paise, 75 paise)* | | | | | | | | | | | | | | | | | ………………*paise* | | | | | | | | | | | |
|  |  | | | | | | | | | |  | | | | | | | |  | | | | | | |  | | | |
| J3 | Did you do anything to treat the illness at home? | | | | | | | | | | Yes 🞎 | | | | | | | | No 🞎 | | | | | | | ***go to J6*** | | | |
| J4 | What did you do? | | | | …………………………………………………………………………………………………………..…………………………………………………………………………… | | | | | | | | | | | | | | | | | | | | | | | | |
| J5 | How long was it from the time you recognized the baby had a problem until you first treated it at home? | | | | | | | | | | | | | | | | | | | | | | | | | | | | |
|  |  | | | _ _ *mins* or _ _ *hours* or _ _ *days* | | | | | | | | | | | | | | | | | | | | | | | | | |
| J6 | Did you seek care from someone outside the home? | | | | | | | | | | | | | | | | Yes 🞎 | | | | | No 🞎 | | | | | | | |
|  | ***If they did not seek care for the baby from someone outside the home go to K1*** | | | | | | | | | | | | | | | | | | | | | | | | | | | | |
| J7 | Where did you seek help? | | | | | | | | | | | | | | | | | | | | | | | | | | | | |
|  | Health Post 🞎 | Maternity home 🞎 | | | | | | | General hospital 🞎 | | | | | | | | | | | | Very large hospital 🞎 | | | | | | | | |
|  | Private facility 🞎 | Other 🞎 | | | | | | | Government hospital 🞎 | | | | | | | | | | | | Outside Mumbai 🞎 | | | | | | | | UHC 🞎 |
|  | Name and location of institution …………………………………………………………….. | | | | | | | | | | | | | | | | | | | | | | | | | | | | |
|  | ***If mother and baby were already in hospital go to next section*** | | | | | | | | | | | | | | | | | | | | | | | | | | | | |
| J8 | How long was it from the time you recognised the baby had a problem until the baby was treated? | | | | | | | | | | | | | | | | | | | | | | | | | | | | |
|  |  | | | | | | | _ _ *mins* or _ _ *hours* or _ _ *days* | | | | | | | | | | | | | | | | | | | | | |
| J9 | Was the baby referred or transferred for further treatment? | | | | | | | | | Sent from gate/OPD 🞎 | | | | | | | | | | Transferred 🞎 | | | | | | | No 🞎 ***Go to J12*** | | |
| J10 | Did you go to the place to which they referred or transferred you? | | | | | | | | | | | | | | | | | | | | Yes 🞎 | | | | | | No 🞎 ***Go to J12*** | | |
| J11 | Where did you go? | | | | | | | | | | | | | | | | | | | |  | | | | | |  | | |
|  | Health Post 🞎 | | | | | Maternity home 🞎 | | | | | | | General hospital 🞎 | | | | | | | | | | | Very large hospital 🞎 | | | | | |
|  | Private facility 🞎 | | Other 🞎 | | | | (what) ……………………………………………. | | | | | | | | | | | | | | | | | | | | | | |
|  | Name and location of institution …………………………………………………………………….. | | | | | | | | | | | | | | | | | | | | | | | | | | | | |
|  |  | | | | | | | | | | | | | |  | | | | | | | |  | | | | | | |
| J12 | Did you seek care from someone else? | | | | | | | | | | | | | | | Yes 🞎 | | | | | | | | No 🞎 | | | | | |
|  | ***If she did not seek care from someone else go to J15*** | | | | | | | | | | | | | | | | | | | | | | | | | | | | |
| J13 | Where did you seek help? | | | | | | | | | | | | | | | | | | | | | | | | | | | | |
|  | Health Post 🞎 | Maternity home 🞎 | | | | | | | | | | General hospital 🞎 | | | | | | | | | | | | | Very large hospital 🞎 | | | | |
|  | Private facility 🞎 | Other 🞎 | | | | | | | | | | (what) ……………………………………………. | | | | | | | | | | | | | | | | | |
|  | Name and location of institution …………………………………………………………………….. | | | | | | | | | | | | | | | | | | | | | | | | | | | | |
| J14 | How long was it from the time you recognised the baby had a problem until the baby was treated? | | | | | | | | | | | | | | | | | | | | | | | | | | | | |
|  |  | | | | | | | _ _ *mins* or _ _ *hours* or _ _ *days* | | | | | | | | | | | | | | | | | | | | | |
| J15 | If your baby had the problem again, where would you go? | | | | | | | | | | | | | Same place 🞎 | | | | | | | | | Other place 🞎 | | | | | Nowhere 🞎 | |

|  | **Section K: Postnatal care** | | | | | | | | | | | | | | | | | |
| --- | --- | --- | --- | --- | --- | --- | --- | --- | --- | --- | --- | --- | --- | --- | --- | --- | --- | --- |
| K1 | Have you gone for a postnatal check-up since the delivery? | | | | | | | | | | | Yes 🞎 ***Go to K3*** | | | | | No 🞎 | |
| K2 | Why not? ***Do not read out the options****.*  ***Interviewer may tick more than one box. Allow the woman to answer and tick options accordingly.*** | | | | | | | | | | | | | | | | | |
| No problem 🞎  Did not see need 🞎  No time to go 🞎  Family did not allow 🞎 | | Nobody to manage children or home 🞎  Nobody to accompany 🞎  Cost of service 🞎  Too far away 🞎 | | | | | | | | | | Not customary 🞎  Afraid of hospital 🞎  Doctor did not tell to visit 🞎 | | | | | | |
|  | ***If no postnatal care go to K6*** | | | | | | | | | | | | | | | | | |
| K3 | How long after the birth did you go for the postnatal check-up? | | | | | | | | | | | _ _ *days* or _ _ *weeks* | | | | | | |
| K4 | Where did you go for the postnatal check-up? | | | | | | | | | | | | | | | | | |
|  | Health Post 🞎 | | | Maternity home 🞎 | | | | General hospital 🞎 | | | | | | Very large hospital 🞎 | | | | |
|  | Private facility 🞎 | | | Other 🞎 | | | | Government hospital 🞎 | | | | | | Outside Mumbai 🞎 | | | | UHC 🞎 |
|  | Name and location of institution …………………………………………………………………….. | | | | | | | | | | | | | | | | | |
| K5 | How many times did you go for a postnatal check-up? | | | | | | | | | __ *times* | | | | | | | | |
| K6 | Has a health worker visited you at home since the birth? | | | | | | | | | Yes 🞎 | | | | | | No 🞎 ***go to K8*** | | |
| K7 | Who visited you? | | | | | | | | | | | | | | | | | |
|  | BMC CHV 🞎 | | ICDS AWW 🞎 | | | | NGO CHV 🞎 | | | Specify name of NGO/CBO 🞎 …… | | | | | | | | |
| K8 | Women have certain problems after a delivery. For example, she may have problems passing urine or with breastfeeding. What sort of problems have you had since the birth? ***Interviewer may tick more than one box*** ***Do not read out the options. Tick options depending on what respondent says*** | | | | | | | | | | | | | | | | | |
|  | No problem at all  | | | | ***go to L1*** | | | | | | | | | | | | | |
|  | Convulsion, fit, seizure, loss of consciousness 🞎  Severe abdominal pain 🞎  Persistent vaginal bleeding 🞎  Leaking of urine or faeces 🞎  Womb coming down or out 🞎  Exhausted, very tired, weak 🞎  Headaches, dizziness 🞎  Nausea, bloating, indigestion 🞎  Constipation 🞎  Cramps 🞎  Backache 🞎  Difficulty seeing at night 🞎  Low or high BP 🞎  Uterus prolapse 🞎 | | | | Breathless while doing normal household tasks 🞎  Jaundice 🞎  Burning when passing urine 🞎  Frequent need to pass urine 🞎  Abnormal vaginal discharge 🞎  Fever 🞎  Diarrhoea 🞎  Cough 🞎  Breast problem: cracked nipple, abscess, engorgement 🞎  Problem with stitches (pain, tear, pus) 🞎  Problems with Caesar stitches 🞎  Afraid of hospital of hospital 🞎  Anaemia 🞎 | | | | | | | | | | | | | |
|  | Other 🞎 | | | | (specify) …………………………………….. | | | | | | | | | | | | | |
|  | ***If only one problem go to K11*** | | | |  | | | | | | | | | | | | | |
| K9 | Which one was the most dangerous and in a paisa scale how you are going to rate it? *(25 paise, 50 paise, 75 paise)* | | | | | | | | | | | | | ………………*paise* | | | | |
| K10 | Did you do anything to treat the illness at home? | | | | | | | Yes 🞎 | No 🞎 | | | | | ***go to K13*** | | | | |
| K11 | What did you do? | | | | | | | …………………………………………………… | | | | | | | | | | |
| K12 | How long was it from the time you recognized you had a problem until you first treated it at home? | | | | | | | | | | | | | | | | | |
|  | _ _ *mins* or _ _ *hours* or _ _ *days* | | | | | | | | | | | | | | | | | |
| K13 | Did you seek care from someone outside the home? | | | | | | | | | | Yes 🞎 | | | | No 🞎 | | | |
|  | ***If she did not seek care from someone outside the home go to L section*** | | | | | | | | | | | | | | | | | |
| K14 | Where did you seek help? | | | | | | | | | | | | | | | | | |
|  | Health Post 🞎 | | | Maternity home 🞎 | | | | General hospital 🞎 | | | | | | Very large hospital 🞎 | | | | |
|  | Private facility 🞎 | | | Other 🞎 | | | | Government hospital 🞎 | | | | | | Outside Mumbai 🞎 | | | | UHC 🞎 |
|  | Name and location of institution …………………………………………………………………….. | | | | | | | | | | | | | | | | | |
| K15 | How long was it from the time you recognised you had a problem until you got treatment? | | | | | | | | | | | | | | | | | |
|  | _ _ *mins* or _ _ *hours* or _ _ *days* | | | | | | | | | | | | | | | | | |
| K16 | Were you referred or transferred to another hospital for further treatment? | | | | | | | | | | | | | | | | | |
|  | Sent from the gate or OPD 🞎 | | | | | | | Transferred 🞎 | | | | | | | No 🞎 ***go to K19*** | | | |
| K17 | Did you go to the place to which they referred or transferred you? | | | | | | | | | | Yes 🞎 | | | | No 🞎 ***go to K19*** | | | |
| K18 | Where did you go? | | | | | | | | | |  | | | |  | | | |
|  | Health Post 🞎 | | | Maternity home 🞎 | | | | General hospital 🞎 | | | | | | Very large hospital  | | | | |
|  | Private facility 🞎 | | | Other 🞎 | | | | Government hospital 🞎 | | | | | | Outside Mumbai 🞎 | | | | UHC 🞎 |
|  | Name and location of institution …………………………………………………………………….. | | | | | | | | | | | | | | | | | |
| K19 | Did you seek care from someone else? | | | | | | | | | | Yes 🞎 | | | | No 🞎 | | | |
|  | ***If she did not seek care from someone else go to K21*** | | | | | | | | | |  | | | |  | | | |
| K20 | Where did you seek help? | | | | | | | | | |  | | | |  | | | |
|  | Health Post 🞎 | | | Maternity home 🞎 | | | | General hospital 🞎 | | | | | | Very large hospital 🞎 | | | | |
|  | Private facility 🞎 | | | Other 🞎 | | | | Government hospital 🞎 | | | | | | Outside Mumbai 🞎 | | | | UHC 🞎 |
|  | Name and location of institution …………………………………………………………………….. | | | | | | | | | | | | | | | | | |
|  | Health Post 🞎 | | | Maternity home 🞎 | | | | General hospital 🞎 | | | | | | Very large hospital 🞎 | | | | |
|  | Private facility 🞎 | | | Other 🞎 | | | | Government hospital 🞎 | | | | | | Outside Mumbai 🞎 | | | | UHC 🞎 |
|  | Name and location of institution …………………………………………………………………….. | | | | | | | | | | | | | | | | | |
| K21 | How long was it from the time you recognised you had a problem until you got treatment? | | | | | | | | | | | | | | | | | |
|  | _ _ *mins* or _ _ *hours* or _ _ *days* | | | | | | | | | | | | | | | | | |
| K22 | If you had the problem again, would you go to the same place? | | | | | Same place 🞎 | | | Other place 🞎 | | | | Nowhere 🞎 | | | | | |

| *Now if you will permit me, I need to ask some more questions about your relationship with your husband and family.*  *Did your husband or family member ever do any of the following things to you when you were pregnant or since you had the baby:* | | | | | | | | | | | | | |
| --- | --- | --- | --- | --- | --- | --- | --- | --- | --- | --- | --- | --- | --- |
|  |  | *Who?* |  |  |  |  |  |  |  |  |  | *How often?* |  |
|  |  | Husband | Father-in-law | Mother-in-law | Brother-in-law | Sister-in-law | Father | Mother | Sister | Brother | Child |  |  |
| Became jealous or angry if you talked to other men? | 🞎 Yes | 🞎 | 🞎 | 🞎 | 🞎 | 🞎 | 🞎 | 🞎 | 🞎 | 🞎 | 🞎 | 🞎 Often | 🞎 Sometimes |
| Accused you of being unfaithful? | 🞎 Yes | 🞎 | 🞎 | 🞎 | 🞎 | 🞎 | 🞎 | 🞎 | 🞎 | 🞎 | 🞎 | 🞎 Often | 🞎 Sometimes |
| Accused you of misusing or stealing household items? | 🞎 Yes | 🞎 | 🞎 | 🞎 | 🞎 | 🞎 | 🞎 | 🞎 | 🞎 | 🞎 | 🞎 | 🞎 Often | 🞎 Sometimes |
| Said or did something to humiliate you in front of others? | 🞎 Yes | 🞎 | 🞎 | 🞎 | 🞎 | 🞎 | 🞎 | 🞎 | 🞎 | 🞎 | 🞎 | 🞎 Often | 🞎 Sometimes |
| Threatenened to hurt or harm you or someone close to you? | 🞎 Yes | 🞎 | 🞎 | 🞎 | 🞎 | 🞎 | 🞎 | 🞎 | 🞎 | 🞎 | 🞎 | 🞎 Often | 🞎 Sometimes |
| Insulted you and makes you feel bad about yourself? | 🞎 Yes | 🞎 | 🞎 | 🞎 | 🞎 | 🞎 | 🞎 | 🞎 | 🞎 | 🞎 | 🞎 | 🞎 Often | 🞎 Sometimes |
| Threatened to either hurt your children or deny you access to them? | 🞎 Yes | 🞎 | 🞎 | 🞎 | 🞎 | 🞎 | 🞎 | 🞎 | 🞎 | 🞎 | 🞎 | 🞎 Often | 🞎 Sometimes |
| Slap you? | 🞎 Yes | 🞎 | 🞎 | 🞎 | 🞎 | 🞎 | 🞎 | 🞎 | 🞎 | 🞎 | 🞎 | 🞎 Often | 🞎 Sometimes |
| Twisted your arm? | 🞎 Yes | 🞎 | 🞎 | 🞎 | 🞎 | 🞎 | 🞎 | 🞎 | 🞎 | 🞎 | 🞎 | 🞎 Often | 🞎 Sometimes |
| Pulled your hair? | 🞎 Yes | 🞎 | 🞎 | 🞎 | 🞎 | 🞎 | 🞎 | 🞎 | 🞎 | 🞎 | 🞎 | 🞎 Often | 🞎 Sometimes |
| Forcibly took something from you? | 🞎 Yes | 🞎 | 🞎 | 🞎 | 🞎 | 🞎 | 🞎 | 🞎 | 🞎 | 🞎 | 🞎 | 🞎 Often | 🞎 Sometimes |
| Punched you with his fist? | 🞎 Yes | 🞎 | 🞎 | 🞎 | 🞎 | 🞎 | 🞎 | 🞎 | 🞎 | 🞎 | 🞎 | 🞎 Often | 🞎 Sometimes |
| Kicked you? | 🞎 Yes | 🞎 | 🞎 | 🞎 | 🞎 | 🞎 | 🞎 | 🞎 | 🞎 | 🞎 | 🞎 | 🞎 Often | 🞎 Sometimes |
| Dragged you? | 🞎 Yes | 🞎 | 🞎 | 🞎 | 🞎 | 🞎 | 🞎 | 🞎 | 🞎 | 🞎 | 🞎 | 🞎 Often | 🞎 Sometimes |
| Beat you up? | 🞎 Yes | 🞎 | 🞎 | 🞎 | 🞎 | 🞎 | 🞎 | 🞎 | 🞎 | 🞎 | 🞎 | 🞎 Often | 🞎 Sometimes |
| Kicked you or hit you in the stomach? | 🞎 Yes | 🞎 | 🞎 | 🞎 | 🞎 | 🞎 | 🞎 | 🞎 | 🞎 | 🞎 | 🞎 | 🞎 Often | 🞎 Sometimes |
| Hit you with a household object? | 🞎 Yes | 🞎 | 🞎 | 🞎 | 🞎 | 🞎 | 🞎 | 🞎 | 🞎 | 🞎 | 🞎 | 🞎 Often | 🞎 Sometimes |
| Tried to choke you? | 🞎 Yes | 🞎 | 🞎 | 🞎 | 🞎 | 🞎 | 🞎 | 🞎 | 🞎 | 🞎 | 🞎 | 🞎 Often | 🞎 Sometimes |
| Burned you? | 🞎 Yes | 🞎 | 🞎 | 🞎 | 🞎 | 🞎 | 🞎 | 🞎 | 🞎 | 🞎 | 🞎 | 🞎 Often | 🞎 Sometimes |
| Locked you up/tied up or restrain you? | 🞎 Yes | 🞎 | 🞎 | 🞎 | 🞎 | 🞎 | 🞎 | 🞎 | 🞎 | 🞎 | 🞎 | 🞎 Often | 🞎 Sometimes |
| Threatened you with a knife, gun or other weapon? | 🞎 Yes | 🞎 | 🞎 | 🞎 | 🞎 | 🞎 | 🞎 | 🞎 | 🞎 | 🞎 | 🞎 | 🞎 Often | 🞎 Sometimes |
| Forced you to have sexual intercourse with him even when you did not want to? | 🞎 Yes | 🞎 | 🞎 | 🞎 | 🞎 | 🞎 | 🞎 | 🞎 | 🞎 | 🞎 | 🞎 | 🞎 Often | 🞎 Sometimes |
| Forced you to perform sexual acts with him or with others when you did not want to? | 🞎 Yes | 🞎 | 🞎 | 🞎 | 🞎 | 🞎 | 🞎 | 🞎 | 🞎 |  |  |  |  |
| Asked to leave house | 🞎 Yes | 🞎 | 🞎 | 🞎 | 🞎 | 🞎 | 🞎 | 🞎 | 🞎 | 🞎 | 🞎 | 🞎 Often | 🞎 Sometimes |
| Thrown out of the house | 🞎 Yes | 🞎 | 🞎 | 🞎 | 🞎 | 🞎 | 🞎 | 🞎 | 🞎 |  |  |  |  |

| *f yes to any of the above, did it happen more or less during your pregnancy, compared to before your pregnancy?* | | | |
| --- | --- | --- | --- |
| 🞎 More during pregnancy | 🞎 More before pregnancy | 🞎 More after pregnancy | 🞎 No difference |
| *If yes to any of the above, did it happen more or less during your pregnancy compared to after your pregnancy?* | | | |
| 🞎 More during pregnancy | 🞎 More before pregnancy | 🞎 More after pregnancy | 🞎 No difference |

| *If yes to any of the above, did the following ever happen as a result of what your husband or other person did to you during or after your pregnancy?* | | | |
| --- | --- | --- | --- |
| *Tick box if yes* |  |  |  |
| 🞎 Cuts | 🞎 Immobility | 🞎 Broken bones | 🞎 Labour started early |
| 🞎 Bruises | 🞎 Minor burns | 🞎 Broken teeth | 🞎 Vaginal bleeding |
| 🞎 Aches | 🞎 Severe burns | 🞎 Other serious injury | 🞎 Miscarriage |
| 🞎 Severe pain | 🞎 Deep wounds |  |  |
| *If any are ticked ‘yes’:*  *Did you need medical treatment for the injury?* | | | |
| 🞎 Yes | 🞎 No |  |  |
| *Has it affected your ability to care for your baby?* | | | |
| 🞎 Yes | 🞎 No |  |  |

| *Since the baby was born, has your husband or other family member:* | | |
| --- | --- | --- |
| Taken the baby and prevented you from being with him/her? | 🞎 Yes | 🞎 No |
| Prevented you breastfeeding the baby? | 🞎 Yes | 🞎 No |
| Not allowed you enough time to look after the baby? | 🞎 Yes | 🞎 No |

| *Has your husband ever:* | | |
| --- | --- | --- |
| Said he does not accept the baby? | 🞎 Yes | *Describe…* |
| Manhandled the baby? | 🞎 Yes | *Describe…* |
| Prevented you seeking care for the baby? | 🞎 Yes | *Describe…* |
| Threatened to hurt the baby? | 🞎 Yes | *Describe…* |

| *Does your husband:* | | | | |
| --- | --- | --- | --- | --- |
| Drink alcohol? | 🞎 Yes *If yes… how often does he get drunk?* | 🞎 Often | 🞎 Sometimes | 🞎 Never |
| Take drugs? | 🞎 Yes *If yes… how often is he affected?* | 🞎 Often | 🞎 Sometimes | 🞎 Never |
| *If yes to either..* |  |  |  |  |
| Is abuse more likely to happen when he is under the influence of alcohol or drugs? | | | 🞎 Yes | 🞎 No |

| *If there was any abuse…*  *Thinking about what you yourself have experienced among the different things we have been talking about:* | | | | | |
| --- | --- | --- | --- | --- | --- |
| Have you ever told anyone else about this? | |  | | 🞎 Yes | 🞎 No |
| Have you ever tried to seek help to stop the person from doing this again? | | | | 🞎 Yes | 🞎 No |
| From where have you sought help to stop this? | | |  |  |  |
| *Let her answer, then probe for other sources of help…* | | |  |  |  |
| *Tick box if yes* |  | |  |  | |
| Maternal family 🞎 | Husband’s family 🞎 | | Panchayat 🞎 | Male friend 🞎 | |
| Female friend 🞎 | Neighbour 🞎 | | Religious leader 🞎 | Dr or medical personnel 🞎 | |
| Police 🞎 | Lawyer 🞎 | | Helpline 🞎 |  | |
| Women’s group 🞎 | *Which…* | | | | |
| Social service organisation 🞎 | *Which…* | | | | |
| Other 🞎 | *Which…* | | | | |

|  | **Section L: Grievance and community groups** | | | | | | | | | |
| --- | --- | --- | --- | --- | --- | --- | --- | --- | --- | --- |
| L1 | During your medical care for pregnancy or delivery, were there any things which you like?  ***Interviewer may tick more than one box. Allow the woman to answer and tick options accordingly.*** | | | | | | | | | |
|  | No 🞎 | | | |  | | | | | |
|  | Guidance on where to go in the hospital 🞎  No queue 🞎  Doctor gave enough time for consultation 🞎  Doctor or nurse explained enough about problems, treatment or referral 🞎  Sheets, laundry, clothes were good 🞎 | | | | Doctor or nurse was available or came when they were needed 🞎  Did not had to pay much for care 🞎  Did not have to wait for long for attention 🞎  Doctor or nurse listened to her 🞎  Food was good 🞎  Hygiene, bathrooms, water were good  Doctor or nurse was polite/nice to her 🞎 | | | | | |
|  | Other 🞎 (specify)*……………………………………………………………………………………………….* | | | | | | | | | |
| L2 | During your medical care for pregnancy or delivery, were there any things about the way you were treated with which you were unhappy? Did you have any disappointing experiences with medical services?  ***Interviewer may tick more than one box. Allow the woman to answer and tick options accordingly.*** | | | | | | | | | |
|  | No 🞎 | | |  | | | | |  | |
| No guidance on where to go in the hospital 🞎  Long queue 🞎  Refused delivery at facility 🞎  Doctor did not give enough time for consultation 🞎  Doctor or nurse did not explain enough about problems, treatment or referral 🞎  Sheets, laundry, clothes were not good 🞎  Doctor or nurse was not available or did not come when they were needed 🞎  Had to pay too much for care 🞎 | | | | Had to wait a long time for attention 🞎  Turned away and told to return later 🞎  Transferred to another facility 🞎  Doctor or nurse did not listen to her 🞎  Food was not good 🞎  Hygiene, bathrooms, water were not good  Doctor or nurse scolded, abused, shouted at or slapped her 🞎 | | | | | | |
|  | Other 🞎 | | | (specify) ………..…………………………….. | | | | | | |
| L3 | What did you do about it? | | | | | Nothing 🞎 | | | |  |
|  | Something 🞎 *what?....* | | | | |  | |  | |  |
| L4 | Did you register a complaint at the health facility? | | | | | Yes 🞎 | | No 🞎 | | ***go to L6*** |
| L5 | Was there any follow-up from the facility about your complaint? | | | | | Yes 🞎 | | No 🞎 | |  |
| L6 | Have you heard of any groups in your basti? | | | | | Yes 🞎 | | No 🞎 | |  |
| L7 | Which groups have you heard of? | | …………………………………………………………………………………………………………………………………………………………………………………………………………………………………………………………………………………………………………………… | | | | | | | |
| L8 | During your pregnancy, delivery and after birth, have your got any help by any community group or group member? | | | | | | | | | |
| Yes 🞎 | | No 🞎 | | | | |  | | | |
| Specify name of community group  | | ………………………………………………………….. | | | | | | | | |

| L9 | What help did you get?  …………………………………………………….………………………………………….  ………………………………………………. | | ***Help on antenatal care*** 🞎  ***Help on delivery care*** 🞎  ***Help on postnatal care*** 🞎  ***Help on newborn care*** 🞎  ***Help on care for mother’s illness*** 🞎  ***Help on care for baby’s illness*** 🞎 | | |
| --- | --- | --- | --- | --- | --- |
| L10 | Are you a member of any group in your basti? | | Yes 🞎 | No 🞎 | ***Stop here*** |
| L11 | Which group? | …………………………………………………………………………… | | | |

***Thank you very much for taking the time to answer our questions.***

***Is there anything you would like to ask?***

| *Interviewer’s comments* |  |
| --- | --- |

**Interview end time** _ _ _ _ **Interview duration** _ _ _ _ *minutes*
